# Optically-controlled bacterial metabolite for cancer therapy

**DOI:** 10.1038/s41467-018-03233-9

**Published:** 2018-04-26

**Authors:** Di-Wei Zheng, Ying Chen, Zi-Hao Li, Lu Xu, Chu-Xin Li, Bin Li, Jin-Xuan Fan, Si-Xue Cheng, Xian-Zheng Zhang

**Affiliations:** Key Laboratory of Biomedical Polymers of Ministry of Education, Department of Chemistry, Wuhan University, Wuhan, 430072 China

## Abstract

Bacteria preferentially accumulating in tumor microenvironments can be utilized as natural vehicles for tumor targeting. However, neither current chemical nor genetic approaches alone can fully satisfy the requirements on both stability and high efficiency. Here, we propose a strategy of “charging” bacteria with a nano-photocatalyst to strengthen their metabolic activities. Carbon nitride (C_3_N_4_) is combined with *Escherichia coli* (*E. coli*) carrying nitric oxide (NO) generation enzymes for photo-controlled bacterial metabolite therapy (PMT). Under light irradiation, photoelectrons produced by C_3_N_4_ can be transferred to *E. coli* to promote the enzymatic reduction of endogenous NO_3_^–^ to cytotoxic NO with a 37-fold increase. In a mouse model, C_3_N_4_ loaded bacteria are perfectly accumulated throughout the tumor and the PMT treatment results in around 80% inhibition of tumor growth. Thus, synthetic materials-remodeled microorganism may be used to regulate focal microenvironments and increase therapeutic efficiency.

## Abstract (web summary)

Targeting tumors with bacteria as vehicles for metabolite therapy suffers from low efficiency and robustness. Here, the authors combine carbon nitride with nitric oxide generation enzyme-positive *E. coli* for photo-controlled metabolite therapy (PMT) and observe increased effects both in vitro and in tumor-bearing mice.

## Introduction

Recently, bacteria-mediated treatments have attracted great attention, especially for tumor therapy due to the exceptional tumor colonizing ability of some bacteria^1,2^. Beyond utilizing their tumor targeting ability, chemical explorations have been made to directly conjugate drug loaded nanomaterials to tumor-targeting bacteria such as *Salmonella* and *Magnetococcus*^3–5^. As a biological strategy, genetic engineering was also used to program bacteria for achieving in situ anti-cancer agents synthesis^6–8^. However, both these strategies have their own intrinsic drawbacks. The limited carrying capacity of chemical approaches and the loss of transgene expression in biological strategy led to insufficient dosage of anti-cancer agents and unsatisfactory therapeutic efficiency^9^. Clearly, neither synthetic nor genetic approaches alone can fully satisfy the current requirements on both stability and high efficiency.

Some bacteria can spontaneously metabolize nontoxic compounds to antineoplastic products (e.g., NO_3_^–^ to NO)^10,11^. However these natural bioreactions are usually too feeble to realize satisfactory therapeutic effects. As we know, some bacteria possess the capacity of driving intracellular reaction at the expense of exogenous electrons. Inspired from this phenomenon, we conceive an idea that charging bacteria with abundant exogenous electrons could help control their intrinsic metabolic activities and boost their latent anti-cancer potential. Recently, numerous nano-sized photocatalytic materials such as CdS and C_3_N_4_ have attracted considerable attention for their photoelectric converting ability, which could continuously transfer light into electric energy^12–14^. These kind of materials not only “charge” bacteria to strengthen their metabolic activities, but also bypass loading limits to realize abundant anti-cancer agents production. This universal strategy might construct a biotic/abiotic loop that allows the photo-induced synthesis of NO from NO_3_^–^ possible.

Inspired by the above photo-induced reduction, we have fused photocatalytic systems with tumor targeted bacteria, and thus to obtain a biotic/abiotic hybrid for light-controlled NO generation. In support of this goal, *E. coli* MG1655, a non-pathogenic bacterium with both tumor targeting and nitrate/nitrite reductase expression, is chosen for modification^15^. First, we synthesize carbon-dot doped carbon nitride (CCN) with suppressed free radical generation capability to achieve in situ photoelectric conversion^16^. Furthermore, CCN and *E*. *coli* are assembled through electrostatic interactions to obtain CCN@*E. coli*. Herein, we propose a concept of photo-controlled bacterial metabolite therapy (PMT), which utilizes modified CCN@*E. coli* to metabolize NO_3_^–^ to antineoplastic NO for cancer treatment under photo-irradiation. We also verify its detailed mechanism by using isotope labeling method and proteomics study. In mammals, endogenic NO is enzymatically produced from l-arginine by nitric oxide synthase. During its physiological processes, NO could be spontaneously oxidized into NO_3_^–^, which is useless to mammalian cells. However, NO_3_^–^ is the primary nitrogen source of the PMT system^17^. Thus, the irreversible NO generation is transformed into circular reaction that maximizes the bioavailability of NO. In all, PMT, which optimizes the biomedical applications of biotic/abiotic hybrid systems, creates a paradigm shift in the way of bacterial cancer treatment.

## Results

### Characterization and mechanism of PMT system

**Fig. 1:**
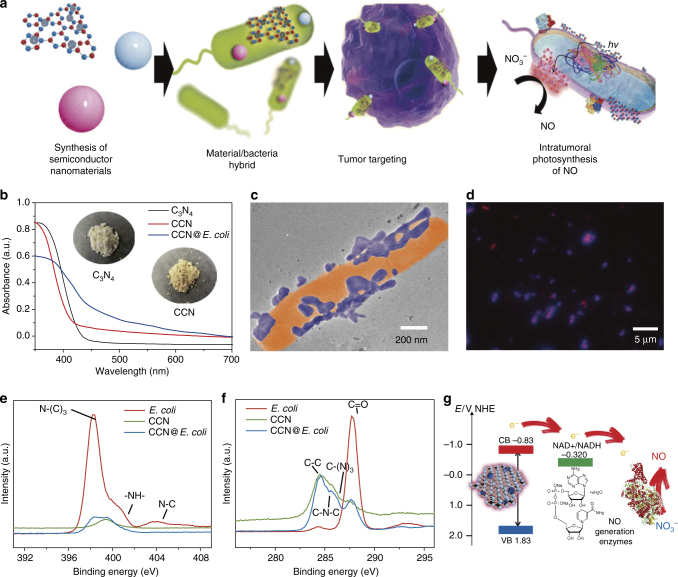
Characterization of PMT system. **a** Schematic diagram of the preparation of PMT system. **b** UV−Vis absorption spectra of as-prepared CCN. **c** TEM image of CCN@*E. coli*. **d** Spinning disk confocal microscope image of CCN@*E. coli* (Blue: CCN; Red: *E*. *coli*). **e** XPS de-convoluted spectra for the N1s orbitals of *E. coli*, CCN, and CCN@*E. coli*. **f** XPS de-convoluted spectra for the C1s orbitals of *E. coli*, CCN and CCN@*E. coli*. **g** Schematic illustration for the photoelectron transport among CCN, electron acceptor and NO generation enzymes

The preparation of CCN@*E. coli* is schematically illustrated in Fig. 1a. As shown in Fig. 1b, C_3_N_4_ doped with carbon dots produced a red shift of the long-wavelength absorption band^18^. The as-prepared CCN was assembled with *E. coli* through electrostatic interactions. TEM image revealed that after the modification, CCNs were positioned on the surface of *E. coli*, and no morphology change of *E. coli* occurred during this process (Fig. 1c). As shown in Fig. 1d, spatial overlap between CCN and *E. coli* (with a Mander overlap coefficient of 0.94) also indicated the successful assembly of CCN@*E. coli*. Appearances of C-(N)_3_ and N-(C)_3_ in X-ray photoelectron spectra (XPS) also demonstrated the successful modification (Fig. 1e, f, Supplementary Figs. 1–4 and Supplementary Note 1).

**Fig. 2:**
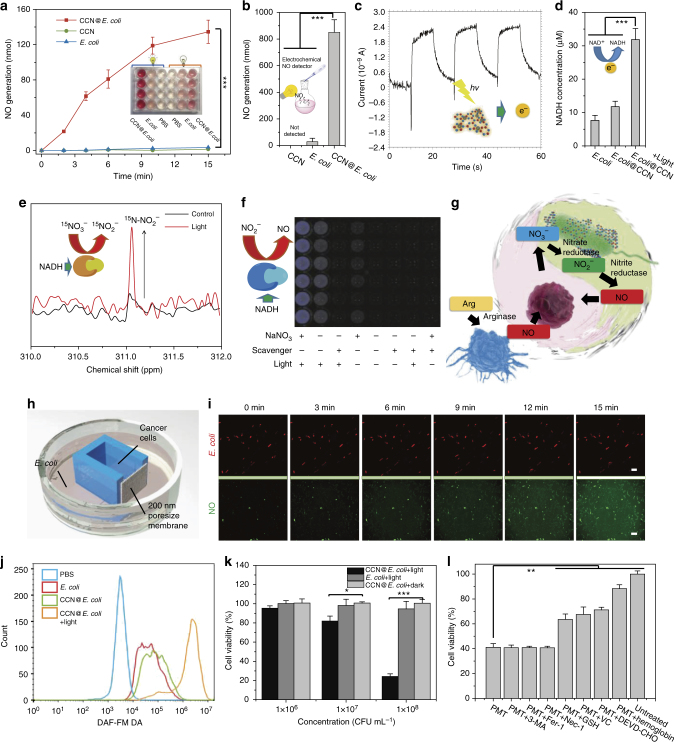
In vitro study of PMT system. **a** Griess method for quantitative determining the NO generation of CCN@*E. coli*, *E. coli* (10^9^ CFU, 2 mL), and CCN. **b** Electrochemical method for monitoring the cumulative NO in the gas phase produced by CCN@*E. coli* after 15 min of irradiation (10^11^ CFU, 5 mL). **c** Transient photocurrent responses of CCN. **d** Intracellular NADH level of wide-type *E. coli*, CCN@*E. coli* with or without light irradiation. **e** ^15^N-NMR for monitoring the in situ CCN@*E*. *coli* NO_3_^−^ metabolism with or without light irradiation. The chemical shift of ^15^N-pyridine in neutral media was defined as 0 ppm. **f** Luminol chemiluminiscence assay for qualitative determining NO generation of CCN@*E. coli*, *E. coli* and CCN. **g** Schematic illustration for the optically controlled NO metabolism close loop. **h** Schematic diagram of the 3D-printing co-culture system. **i** Images from the co-culture system time series sequentially observing CCN@*E. coli* movement and NO generation (Scale bar: 2 μm for the first row and 100 μm for the second row). **j** Flow cytometry for measuring the intracellular NO concentration of 4T1 cells after various treatment. **k** Cell viability of 4T1 cells co-cultured with CCN@*E. coli*. **l** Cell viability of 4T1 cells co-cultured with CCN@*E. coli* after 3-MA (3-Methyladenine, autophagy inhibitor), Fer-1 (ferrostatin-1, ferroptosis inhibitor), Nec-1 (Necrostatin-1, necroptosis inhibitor), Ac-DEVD-CHO (apoptosis inhibitor), hemoglobin treatment (NO scavenger), glutathione (GSH, ROS trapper), sodium ascorbate (VC, ROS trapper). Significance between every two groups was calculated using unpaired two-tailed Student’s *t*-test unless otherwise indicated. *P < 0.05, **P < 0.01, ***P < 0.001. The mean values and S.D. are presented. Data of panels **a**, **b**, **d**, **f**, **j**, **k**, and **l** are from >4 biological replicates per sample

We speculate that due to its suitable band gap, photoelectrons excited from CCN are able to be transferred to NO generation enzymes in *E. coli* via electron carriers (e.g., NADH, reduced nicotinamide adenine dinucleotide)^19^. Subsequently, NO_3_^–^ may be enzymatically reduced to NO in a NADH-dependent manner (Fig. 1g)^20^. Herein, the optically controlled NO release behavior of CCN@*E. coli* was studied following the classic Griess method (Fig. 2a). Within 15 min, the NO concentration reached 33 μM, which was sufficient to induce the apoptosis of cancer cells^21^. In contrast, neither CCN nor *E. coli* alone could produce any NO under light irradiation. Besides, decreased NO_3_^–^ content was also observed upon CCN@*E. coli* treatment, and this phenomenon indicated that NO was indeed transformed from nitrate in the medium (Supplementary Fig. 5a). By using an electrochemical NO sensor, cumulative NO in the gas phase was also detected, and this result clearly demonstrated the NO generation capacity of CCN@*E. coli* (Fig. 2b).

To study the NO generation mechanism of CCN@*E. coli*, we firstly confirmed the photo-excited electron generation of as-prepared CCN. As shown in Fig. 2c, reproducible photocurrent response under on-off light cycle demonstrated the excellent stability of CCN. During the photo-induced reaction, the intracellular NADH concentration of CCN@*E. coli* increased by 4.4 times, and this result indicated that *E. coli* utilized photogenerated electrons from illuminated CCN to carry out intracellular reactions (Fig. 2d and Supplementary Fig. 5b).

After the successfully demonstration of material-cell electron transfer process, we further investigated the intracellular conversion of electric energy to chemical energy. To trace the metabolism of NO, Na^15^NO_3_ labeled by ^15^N isotope was used as a nitrogen source, and in situ ^15^N nuclear magnetic resonance (^15^N-NMR) spectroscopy was utilized to study ^15^N metabolites in the PMT system^22^. As shown in Fig. 2e, after the light irradiation, a strong peak at 311.1 ppm appeared, which could be attributed to ^15^NO_2_^–^ as an intermediate product of NO generation. However, in the absence of light, only an extremely trace level of ^15^NO_2_^–^ was found. Further, to specifically detect the generation of NO, a luminol-hydrogen peroxide chemiluminescence system was used^23^. As presented in Fig. 2f, the NO generation of CCN@*E. coli* was NO_3_^–^ and light-dependent. Carboxy-PTIO, a specific scavenger of NO but not of NO_2_^–^, was found to strongly suppress the luminescence intensity. Besides, quantitatively, sodium tungstate (inhibitor of nitrate reductase) and sodium azide (inhibitor of nitrite reductase) exhibited significant suppression by 77% and 73% of luminescence, respectively. Then, the ultraviolet (UV) oxy-hemoglobin spectrophotometry was also used to qualitatively demonstrate the generation of NO (Supplementary Fig. 5b). And this result implied the light-controlled NO generation ability of CCN@*E. coli*.

Under normal physiological condition, l-arginine is transformed into NO by nitric oxide synthase positive cells such as macrophages. However, since the source is limited and NO could be finally oxidized to nontoxic NO_3_^–^, the anti-cancer potential of the physiologically produced NO is blunted. During PMT treatment with the assistance of CCN@*E. coli*, the irreversible intratumoral NO metabolism was converted into a circular reaction, which maximized the bioavailability of NO. The mechanism of PMT system is a two-step process. First, *E. coli* utilized photogenerated electrons from illuminated CCN to carry out the endogenous NO_3_^–^ reduction and NO generation (Fig. 2g). Then, cell apoptosis was triggered by the as-produced NO. To prove the extensive applicability of this mechanism, combinations between other photoelectric nanomaterials and nitrate/nitrite reductase positive bacteria were also tested. Herein, assemblies of CdS nanoparticles/*E. coli* and CdS nanoparticles/*Bacillus subtilis* were prepared and their NO producing abilities were also studied. As presented in Supplementary Fig. 6 and Supplementary Fig. 7, we discovered that these hybrid systems also exhibited the capacity of photo-induced NO generation (discussed in Supplementary Note 2).

### In vitro anti-cancer study of PMT

To allow metabolite exchange between cancer cells and bacteria without direct contact, we fabricated a co-culture device by using stereolithography three-dimensional (3D) printing technology (Fig. 2h and Supplementary Fig. 8). In this culture system, NO could diffuse through the porous membrane from CCN@*E. coli* (cultured in the outer chamber) to cancer cells (cultured in the inner chamber), whereas, neither CCN@*E. coli* nor cancer cells could migrate through this membrane. Herein, diaminofluorescein-FM diacetate (DAF-FM DA) was used to measure the intracellular NO concentration^24^. As shown in Fig. 2i, improved green fluorescence in 4T1 cells indicated that NO produced from PMT system could effectively diffuse to the nearby chamber and upregulate the NO level in cancer cells. Neither wide-type *E. coli* nor CCN@*E. coli* without light irradiation could increase the intracellular NO concentration (Fig. 2j).

As shown in Fig. 2k, the cell viability was significantly suppressed when giving with light-irradiated CCN@*E. coli*, and up to 70% of 4T1 cells could be killed within 24 h in a CCN@*E. coli* dose of 10^8^ CFU mL^−1^. Moreover, hemoglobin, a scavenger of NO, and Ac-DEVD-CHO, an inhibitor of apoptosis were found to effectively rescue 4T1 cells from apoptosis. Meanwhile, necrostatin-1 (inhibitor for necroptosis), 3-methyladenine (inhibitor for autophagy), and ferrostatin-1 (inhibitor for ferroptosis) could hardly rescue 4T1 cells from death (Fig. 2l). Based on the above observations, we could deduce that apoptosis induced by NO was the main cause of death for 4T1 cells^25^. Besides, we also confirmed that the death of cancer cells was associated with the increased oxidative stress level (Supplementary Fig. 9, Supplementary Fig. 10, and Supplementary Note 3), thus ROS traps including glutathione and sodium ascorbate could also reduce the cytotoxicity of PMT towards cancer cells. Since MG1655 is a non-pathogenic bacterium, directly cultured with MG1655 did not cause significant cytotoxicity in 4T1 cells (Supplementary Fig. 11).

### In vivo bio-distribution study of PMT system

**Fig. 3:**
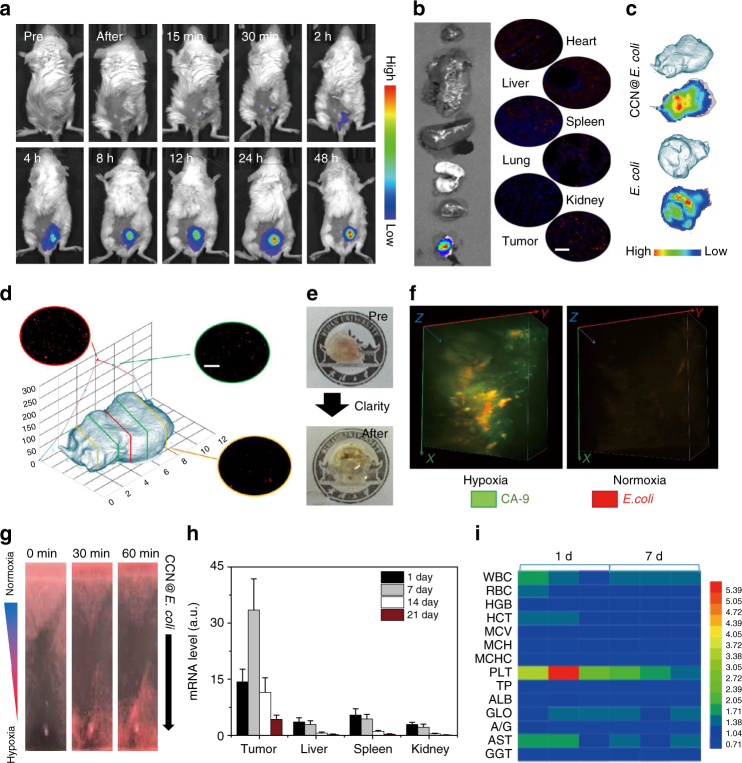
In vivo bio-distribution and bio-compatibility of PMT system. **a** In vivo fluorescence imaging of tumor-targeting ability of CCN@*E. coli*. The imaging was performed at different time points after the i.v. injection of CCN@*E. coli* (10^8^ CFU mL^−1^ in 200 μL saline, *n* = 5). **b** Ex vivo fluorescence imaging for investigating the bio-distribution of CCN@*E. coli* (Scale bar: 100 μm). **c** Tumor accumulation of *E. coli* and CCN@*E. coli*. **d** Transverse sections of CCN@*E. coli* treated tumor after targeting and penetrating (Scale bar: 100 μm). The experiment was performed at 24 h after the i.v. injection of CCN@*E. coli* (10^8^ CFU mL^−1^ in 200 μL saline, *n* = 3). **e** Images of whole mouse tumor and CLARITY technique treated optically transparent tumor. The experiment was performed at 24 h after the i.v. injection of CCN@*E. coli* (10^8^ CFU mL^−1^ in 200 μL saline). **f** Three-dimensional fluorescence imaging for visualizing the co-localization of CCN@*E. coli* and hypoxic region within the tumor. **g** In vitro hypoxia-induced chemotaxis of CCN@*E. coli*. **h** PCR analysis of CCN@*E. coli* within liver, spleen, and kidney decreased with the time prolonging. The PCR analysis was performed on the 1st, 7th, 14th, and 21th days after the i.v. injection of CCN@*E. coli* (10^8^ CFU mL in 200 μL saline, *n* = 10). The mean values and S.D. are presented. **i** Blood biochemistry and hematologic indexes of mice after i.v. injection with CCN@*E. coli*. The experiment was performed on the 1st and 7th days after the i.v. injection of CCN@*E. coli* (10^8^ CFU mL^−1^ in 200 μL saline, *n* = 3)

To evaluate the tumor-targeting ability of PMT system, DIR labeled CCN@*E. coli* was i.v. injected into 4T1 tumor-bearing mice. As shown in Fig. 3a, stronger fluorescence was detected within the tumor position with the time prolonging. As shown in Fig. 3b, ex vivo fluorescence imaging revealed that a massive amount of material accumulated at the tumor position, whereas, negligible liver or kidney retention could be found. It should be noticed that the modification of CCN did not disturb the tumor-targeting ability of *E. coli* (Fig. 3c).

Commonly, for their diffusion limits, most of synthetic drug carriers could hardly penetrate tumor tissue. We speculated that PMT system with the propelling force may be able to reach the deeper region of the xenografts. Herein, histological sections of the tumor at different depths revealed that the PMT system were distributed among tumor tissue (Fig. 3d and Supplementary Fig. 12)^3^. Interestingly, we found that more bacteria were accumulated at the center of the xenograft. And we deduced that the satisfactory tumor penetrability of CCN@*E. coli* might come from the hypoxia-mediated chemotaxis of facultative anaerobic *E. coli*. To comprehensively reveal the tumor penetrability feature of CCN@*E. coli*, the surfactant assistant tissue clearing technology, namely CLARITY, was used to convert tumor tissue into transparent form (Fig. 3e)^26^. Then, carbonic anhydrase-IX (CA-9), a biomarker that highly correlated with tumor hypoxia was used to stain the tumor hypoxic region. From 3D tumor fluorescence images, co-localization of red fluorescence from CCN@*E. coli* and green fluorescence from CA-9 was observed, whereas, CCN@*E. coli* fluorescence could be found in normoxic regions (Fig. 3f). The same result also obtained by the 2D immunofluorescence image (Supplementary Fig. 13 and Supplementary Note 4). As illustrated in Fig. 3g, CCN@*E. coli* was found to spontaneously swim from normoxic region to hypoxic region along an established O_2_ gradient. Thus, we demonstrated that the PMT system could sufficiently harness the hypoxia-mediated chemotaxis of *E. coli*, and reach the hypoxia region inaccessible for other conventional vectors with high efficiency.

Subsequently, we studied the clearance kinetics of CCN@*E. coli* in major metabolic organs such as liver, spleen and kidney. Evidenced by both PCR analysis and qualitative immunofluorescent staining, most of the bacteria could be eliminated with the time prolonging. After 3 weeks, negligible amount of bacteria could be detected in immunocompetent mice (Fig. 3h and Supplementary Fig. 14, and Supplementary Fig. 15a). No long-term side effects of PMT could be found from blood biochemistry and hematologic analysis (Fig. 3i). Subsequently, we also verified that the administration of antibiotics could accelerate the clearance of CCN@*E. coli*, and this result proved that the PMT treatment was completely controllable in patients’ body (Supplementary Fig. 15a). Mice body temperature and white blood cell (WBC) counts after the treatment were also monitored (Supplementary Fig. 15b, c). However, neither body temperature up nor WBC count increase was observed, this result indicated that CCN@*E. coli* at a dose of 10^8^ CFU per mouse did not cause sepsis.

### In vivo NO generation of PMT system

**Fig. 4:**
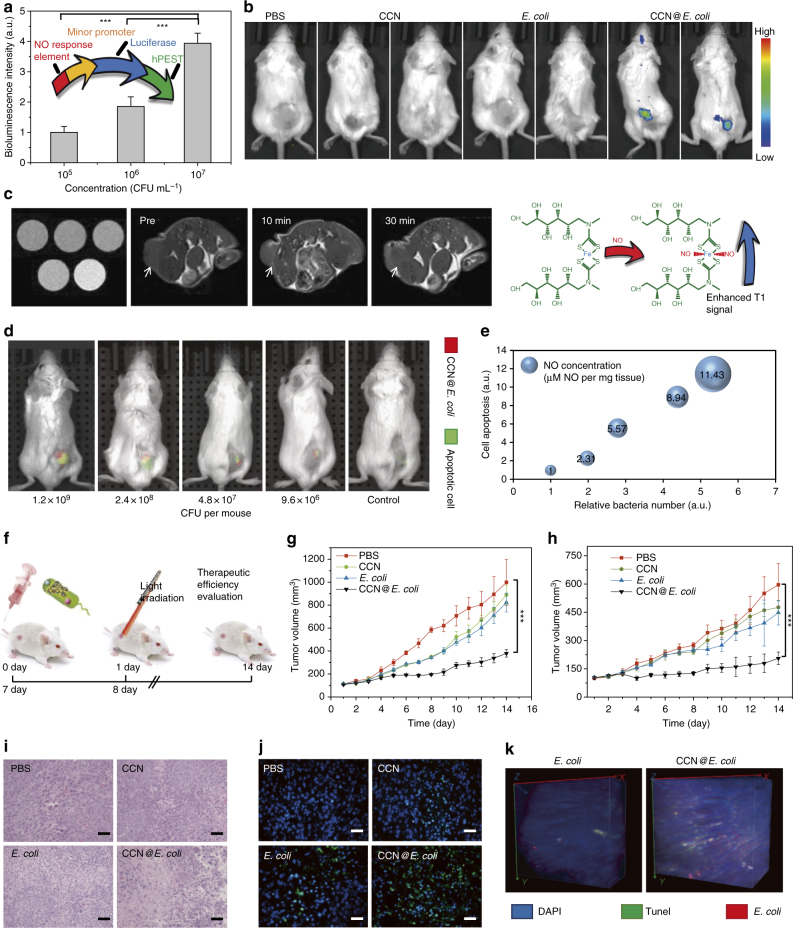
In vivo NO generation and anti-cancer effect of PMT system. **a** Schematic illustration of the NO responsive element contained sensing plasmid and its in vitro NO responsiveness (*n* = 3). **b** In vivo NO generation of PMT system in 4T1^Nrf2-luc^ tumor-bearing mice. The imaging was performed 24 h after the i.v. injection of *E. coli*, CCN@*E. coli* (10^8^ CFU mL^−1^ in 200 μL saline), and CCN (5 mg mL^−1^ in 200 μL saline). **c** MRI images for detecting both in vitro and in vivo NO generation with the assistance of NO responsive contrast agent Fe-MGD (*n* = 3). The in vivo imaging was performed 24 h after the i.v. injection of CCN@*E. coli* (10^8^ CFU mL^−1^ in 200 μL saline). **d** Imaging apoptotic response in vivo with annexin V-Cy5.5 (Red: CCN@*E. coli*, Green: apoptotic cancer cells). The in vivo imaging was performed 48 h after the i.v. injection of CCN@*E. coli* (10^8^ CFU mL^−1^ in 200 μL saline). **e** Semiquantitative analysis of relationship among intratumoral NO concentration (evaluated by the circle area), bacteria count, and signal intensity of apoptotic cells (*n* = 3). **f** Schematic diagram of the process of in vivo anti-cancer therapy. Intravenous injection of *E. coli*, CCN@*E. coli* (10^8^ CFU mL^−1^ in 200 μL saline), CCN (5 mg mL^−1^ in 200 μL saline) and phosphate buffer solution treated group (PBS) were performed on the 1st and 7th days (*n* = 6). Light irradiation was performed 24 h after the injection ( > 630 nm, 30 mW cm^−2^, 15 min). **g** In vivo anti-cancer effect of PMT in 4T1 tumor-bearing mice (*n = *6). **h** In vivo anti-cancer effect of PMT in CT26 tumor-bearing mice (*n* = 6). **i** H&E staining of 4T1 tumor after 14 days of PMT (Scale bar: 100 μm). **j** TUNEL staining of 4T1 tumor after 14 days of PMT (Scale bar: 100 μm). **k** Three-dimensional fluorescence imaging for visualizing the co-localization of CCN@*E. coli* and apoptotic region within the tumor. Significance between every two groups was calculated using unpaired two-tailed Student’s *t*-test. *P < 0.05, **P < 0.01, ***P < 0.001. The mean values and S.D. are presented

To further characterize the photosynthetic behavior in vivo, we designed and built a Nrf2 controlled luciferase expression plasmid to probe the in vivo NO generation and 4T1 cells were transfected to build 4T1^Nrf2^ cell for in situ NO detection^27^. As shown in Fig. 4a and Supplementary Fig. 16, in vitro exposure under CCN@*E. coli* resulted in a rapid increase in the bioluminescence of 4T1^Nrf2-luc^ cell. The in vivo efficiency of PMT system was also studied in 4T1^Nrf2-luc^ tumor-bearing Balb/c mice. As shown in Fig. 4b, neither CCN nor *E. coli* alone could induce detectable NO generation. Whereas, the bioluminescence intensity of CCN@*E. coli* treated group was significantly increased. Next, iron/*N*-(dithiocarbamoyl)-*N*-methyl-d-glucamine complex (Fe-MGD), a magnetic resonance imaging (MRI) probe used for NO detection was also synthesized^28^. As shown in Fig. 4c, the enhanced T1 signal within the tumor after the injection of CCN@*E. coli* and light irradiation was observed, which further proved the optically controlled NO generation in vivo.

### In vivo anti-cancer mechanism of PMT

In vivo treatment responses of PMT were also studied by using Cy5.5 conjugated annexin V^29^. As presented in Fig. 4d, with the increase of CCN@*E. coli* injection, more CCN@*E. coli* was accumulated at the tumor position. After light irradiation, comparable annexin V fluorescence increase and NO generation in PMT treated group were found. Semiquantitative analysis in Fig. 4e also proved the positive correlation among intratumoral bacteria count, tumor-cytotoxicity, and NO concentration.

Furthermore, the anti-cancer efficiency of PMT was examined in both 4T1 tumor-bearing mice and CT26 tumor-bearing mice and the therapeutic schedule was shown in Fig. 4f. In 4T1 tumor-bearing mice, PMT treatment inhibited 79.3% of tumor growth (Fig. 4g). Besides, PMT also suppressed 70.2% of tumor growth in CT26 tumor (Fig. 4h and Supplementary Fig. 17a, d). These results indicated that PMT should be appropriate for treating different cancer types. Then, we also noticed that the therapeutic effect of CCN@*E. coli* exhibited a dose-dependent effect, and the tumor inhibition rate increased with the increasing doses of CCN@*E. coli* (Supplementary Fig. 17e, f). H&E and TUNEL staining clearly showed that a PMT treatment led to irreversible cell apoptosis. However, *E. coli* and CCN treatments only displayed very feeble effects (Fig. 4i, j and Supplementary Fig. 18). Three-dimensional fluorescence imaging of apoptotic cancer cells and CCN@*E. coli* distribution (Fig. 4k) indicated that much more apoptotic cancer cells in PMT group could be found. Additionally, it should be noted that the distance between apoptotic cancer cells and CCN@*E. coli* was close in space, but the fluorescence was not completely merged. This phenomenon suggested that cancer cells were eliminated by diffusible NO generated in PMT rather than *E. coli* treatment.

**Fig. 5:**
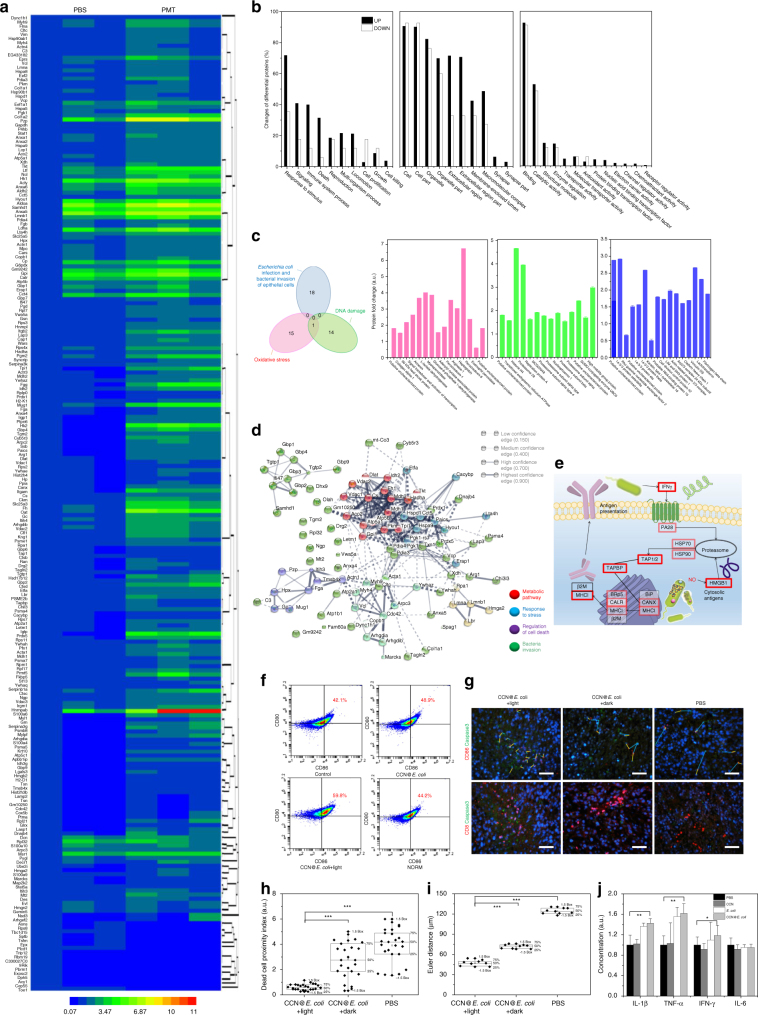
Isobaric tags for relative and absolute quantitation proteomics study for exploring the anti-cancer mechanism of PMT system. **a** Heat map showing significantly upregulated and downregulated proteins (fold change ≥ 1.5 and *P* < 0.05). **b** Biological functions, cellular component, and molecular functions of differential proteins based on GO annotation. **c** Venn diagram indicated the non-overlapped biological effect of *E. coli* and NO generated from PMT system. Histograms of differential proteins associated with *E. coli* infection, bacteria invasion, DNA damage, and oxidative stress based on GO annotation. **d** Functional interaction network of PMT regulated proteins was analyzed by using Search Tool for the Retrieval of Interacting Genes/Proteins (STRING) algorithm. **e** KEGG pathway analysis for antigen processing and presentation associated differential proteins (upregulation proteins were marked with a red box). **f** Flow cytometry for evaluating the DC maturation. **g**–**i** Dead-cell proximity index and Euclidean distance matrix analysis index of CD-86/caspase-3 after PBS or CCN@*E. coli* treatment (*n* = 6, scale bar: 50 μm). **j** Serum IL-1β, TNF-α, IFN-γ, and IL-6 level after PBS, CCN, *E. coli*, or CCN@*E. coli* treatment (*n* = 6). Significance between every two groups was calculated using unpaired two-tailed Student’s *t*-test. *P < 0.05, **P < 0.01, ***P < 0.001. Data are mean ± S.D. (**j**) or median (**h**, **i**) linked with individual values

Afterward, quantitative proteomics technique was used to explore the detailed anti-cancer mechanism of PMT. Herein, 4735 proteins were identified^30^. As shown in Fig. 5a and Supplementary Fig. 19, after PMT treatment, 222 upregulated differential proteins and 17 downregulated differential proteins within tumor tissue were determined (fold change ≥ 1.5 and *P* < 0.05, hypergeometric test). Results of both cluster analysis and principal component analysis revealed the significant differences between phosphate buffer solution (PBS) and PMT treatment groups. On the basis of the Gene Ontology (GO) analysis on differential proteins in biological processes, we found that proteins that respond to stimuli, signals, cell death, immune system and cell killing were more upregulated in PMT group (Fig. 5b). In contrast, more downregulations were observed in cell proliferation and growth-related proteins. These data evidenced that PMT induced a high stress level in tumor, which was ascribe to the oxidative damage induced by NO. As a proof, proteins associated with antioxidant activity also obviously decreased. Commonly, the cytotoxicity of NO towards cancer cells was mainly attributed to its capacity of inducing oxidative stress and initiating DNA damage. The Venn diagram shown in Fig. 5c indicated that there was no overlap between *E. coli* infection/invasion related proteins and DNA damage/oxidative stress associated proteins. Thus, the proteomics study suggested that the direct cytotoxicity of CCN@*E. coli* towards cancer cells should be mainly attributed to NO itself.

Interestingly, a significant rise in the proteins level involved in immune responses was found in GO analysis. This phenomenon suggested that immune system might contribute to the anti-cancer effect of PMT in vivo. The same conclusion could also be drawn from protein–protein interactions network analysis after PMT treatment. Four optimal clusters containing differential proteins involved in metabolic pathway responding to stress, regulation of cell stress, and bacteria invasion were identified (Fig. 5d). As shown in Fig. 5e and Supplementary Fig. 20, in Kyoto Encyclopedia of Genes and Genomes (KEGG) pathway analysis, the comprehensive activation of antigen presentation pathway was found. Besides, the upregulation of high-mobility-group box alarmin protein (HMGB), a dominant protein involved in triggering antigen presentation leading to immunogenic cell death, was also affirmed (Supplementary Fig. 21)^31^. All these discoveries revealed that PMT might induce immunogenic cell death via HMGB triggered MHC class I-mediated pathway. In an in vitro simulation environment, PMT treatment was found to induce DCs mutation (Fig. 5f). By using Euler distance and dead-cell proximity index analysis, both DC and CD8 + cytotoxicity T cells immune activation was verified (Fig. 5g–i), and increased concentration of immune cytokines in serum demonstrated that PMT provoked this immune response (Fig. 5j and detailed discussed in Supplementary Note 5). Overall, these results uncovered that, apart from directly generating cytotoxic NO, immune response might also play a critical role in the anti-cancer mechanism of PMT. It was reported that, inducing immunogenic cancer cell death relies on two key factors, including antigenicity and adjuvanticity^32^. In this process, NO generated by CCN@*E. coli* could trigger the release of tumor antigens, whereas *E. coli* itself might act as an adjuvant to amplify the immune response^33^. Given the increasing interest in inducing immune response in anti-cancer therapy, it is conceivable that PMT treatment might provide a new strategy to prepare cancer vaccine or cooperate with immune checkpoint therapy^34,35^.

## Discussion

We have developed a biotic/abiotic hybrid system, CCN@*E. coli*, to achieve photo-controlled bacterial metabolite therapy. By combining both selectivity of biosynthetic enzymes and robustness of photocatalytic nanomaterials, the bacterial NO generation could be controlled with light irradiation. In addition, the using of self-powered, non-pathogenic microorganism provided us with an option to overcome shortcomings of classical nanomaterials in tumor targeting and organ penetrating. The NO generation, cytotoxic cell killing effect and mechanism of PMT were studied comprehensively. Interestingly, results obtained from proteomics also suggest that immune response may involve in this therapy, and PMT strategy may lead to useful actuation in cancer immunotherapy. We expect the strategy developed in this study will provide a new approach for the design and preparation of living biomaterials.

## Methods

### Materials

Citric acid, urea, Cd(NO_3_)_2_, NaNO_3_, NaNO_2_, acrylic amide, and cysteine were purchased from Shanghai Reagent Chemical Co., and allyl chloroformate was purchased from TCI Co. NO assay kit, DAF-FM DA assay, DCFH-DA assay, TUNEL assay, and JC-1 assay and hemoglobin were purchased from Beyotime Biotechnology Co. β-Nicotinamide adenine dinucleotide (NADH), β-nicotinamide adenine dinucleotide hydrate (NAD + ) and 2,2′-azobis[2-imidazolin-2-yl)propane] dihydrochloride and paraformaldehyde were purchased from Acros Co. Methyl-glucaminedithiocarbamate (MGD) sodium salt monohydrate was purchased from Santa Cruz Biotechnology Co. MTT cell proliferation assay kit was purchased from Biosharp Co. Nafion (D520) was purchased from DuPont Instrument. Na^15^NO_3_ was purchased from Sigma-Aldrich. Cy5.5 was purchased from AmyJet Scientific Inc. Transwell® was purchased from Corning Costar. Amplite™ NAD/NADH assay kit was purchased from ABD Bioquest Inc. 3-Methyladenine, ferrostatin-1, necrostatin-1, and DEVD-CHO were purchased from Selleck Co. 1,1’-Dioctadecyl-3,3,3’,3’-tetramethylindotricarbocyanine iodide (DIR), Hoechst 33342 and DAPI were purchased from Thermofisher Scientific. Anti-CD11c (Clone N418, 1: 800), anti-CD86 (Clone GL-1, 1:200), anti-CD8 (Clone 53-6.7, 1:500), and anti-CD80 (Clone 16-10A1, 1:500) antibodies were purchased from Biolegend. Anti-HMGB1 (ab79823, 1:1000) and anti-active-caspase-3 (ab2302, 1:1000) antibodies were purchased from Abcam.

### Characterization

Transmission electron microscopy images were obtained from a JEM-2100 (JEOL) transmission electron microscope. FT-IR spectrum was measured on a Spectrum Two Fourier Transform Infrared (FT-IR) Spectrophotometer (Perkin-Elmer). XPS was recorded with an ESCALAB 250Xi photoelectron spectrometer (Thermo Fisher) using monochromatic Al Kα X-ray source. Powder X-ray diffraction (XRD) analysis was performed with a Rigaku MiniFlex 600 X-ray diffractometer using Cu-Kα (*λ* = 1.5418 Å). Zeta potential and particle size were studied by using a zeta sizer (Nano ZS, Malvern Instruments). Ultraviolet and visible diffuse reflectance spectroscopy (UV–Vis DRS) was measured with a Shimadzu UV-3600 UV–Vis spectrophotometer. Immunofluorescence sections were performed on a wide-field fluorescence microscopy (Olympus IX73). Spinning disk confocal microscopy images were obtained on a Perkinelmer UltraVIEW VoX 3D live cell imaging system. Small animal fluorescence imaging was performed with a Perkin-Elmer living image In Vivo Imaging System (IVIS) spectrum. Bioluminescence intensity and optical density were measured with a SpectraMax i3x multi-mode detection platform (Molecular Devices).

### Cell line and bacteria

4T1 cells (ATCC-CRL-2539) and CT26 cells (ATCC-CRL-2638) were purchased from China Center for Type Culture Collection. *Escherichia coli* (*E. coli*, strain MG1655, ATCC-PTA-4823) and *Bacillus subtilis* (*B. subtilis*, ATCC-6051) were obtained from China Center of Industrial Culture Collection Cultures.

### Preparation of CCN@bacteria

CCN (20 mg) was resuspended in 25 mL *E. coli* or *B. subtilis* suspension (1 × 10^7^ CFU mL^−1^, 10 mL), and then placed at 37 °C with shaking (200 rpm) for 4 h. The CCN@bacteria was washed with Luria-Bertani (LB) medium and centrifuged twice (590 ×* g*).

### Preparation of CdS@bacteria

*E. coli* or *B. subtilis* were cultured in LB medium supplemented with 10 mM glucose and 0.1 wt.% cysteine and incubated at 37 °C for 24 h. The culture was then reinoculated at 5.0 vol.% into fresh LB medium with 1 mM Cd(NO_3_)_2_. After 48 h of growth, the CdS@bacteria was centrifuged at 590 ×* g* for 5 min, and resuspended into fresh LB medium to obtain the CdS@bacteria hybrid.

### NO generation measurements

The NO generation of CCN@*E. coli* was measured using Griess reagent. Briefly, 10^9^ CFU of CCN@*E. coli* was cultured in LB medium (2 mL) and exposed under red light ( > 630 nm, 30 mW cm−^2^). At different time, 50 μL of culture medium was collected and added into a 96-well plate. After the cell was lysed, 50 μL of A medium (45 mM p-sulfanilic acid, 5 M acetic acid) was added. Five minutes later, 50 μL of B medium (35 mM α-Naphthylamine, 5 M acetic acid) was added. The absorbance at 540 nm was measured. UV oxy-hemoglobin spectrophotometry was also used for measuring the NO generation. In all, 10^9^ CFU of CCN@*E. coli* was cultured in LB medium (2 mL) and exposed under red light ( > 630 nm, 30 mW cm^−2^) for 15 min. At different time, 300 μL of culture medium was collected and mixed with oxy-hemoglobin (10 μM). The NO generation was measured as the difference between the absorbance at 401 and 421 nm in a dual-wavelength spectrophotometer (Lambda Bio 40, PerkinElmer, USA).

### Monitoring the cumulative NO in the gas phase

Cumulative NO in the gas phase (diffused from CCN@*E. coli* in the solution) was measured with an electrochemical NO sensor (Alphasense, Membrapo, Switzerland). Briefly, 10^11^ CFU of CCN@*E. coli* (5 mL) was placed in a well-sealed bottle (10 mL) and irradiated for 15 min. Then, the NO sensor was used to measure the NO concentration within the bottle.

### Nitrate ion concentration measurement

The nitrate reduction during CCN@*E. coli*-mediated NO generation was measured using an ion chromatography. Briefly, 10^9^ CFU of CCN@*E. coli* was cultured in LB medium (2 mL) and exposed under red light ( > 630 nm, 30 mW cm^−2^) for 15 min. Then, the medium was centrifuged (1660 × *g*, 3 min) and the supernatant was subsequently collected. NO_3_^–^ concentration of the supernatant was measured with an ion chromatography (ICS-2500, Dionex, USA).

### Electrochemical measurement

Photocurrent generated from CCN was measured with an electrochemical analysis system (CHI830, Chenhua Instruments, Shanghai, China) in a standard three-electrode system consisted of a platinum electrode, a saturated calomel electrode (SCE) and a modified GCE (3 mm in diameter) working electrode. An 18 W LED lamp was utilized as the light source. CCN modified electrode was prepared with a common coating method. Briefly, 6 μL of CCN aqueous solution (2 mg mL^−1^) containing 0.5% Nafion (DuPont D520) was dropped on to the GCE and dried at room temperature to obtain CCN coated GCE. For investigating catalytic ability of CCN, 0.1 M Na_2_SO_4_ solution was used as the electrolyte.

### Co-localization study of CCN@*E. coli*

The integrity of the CCN@*E. coli* was determined by the co-localization of the CCN (auto blue fluorescence) and the bacteria (red fluorescence). Then *E. coli* (MG1655-mcherry) were mixed with CCN (2 mg mL^−1^, 10 mL). Subsequently, the bacteria were washed with PBS and fixed with 4% paraformaldehyde for 30 min before imaging under spinning disk confocal microscopy.

### ^15^N-NMR analysis

CCN@*E. coli* (10^9^ CFU mL^−1^) was cultured in a Na^15^NO_3_ contained LB medium (300 mg mL^−1^). Then, CCN@*E. coli* was exposed under light irradiation ( > 630 nm, 30 mW cm^−2^, 15 min). Two hours after the irradiation, the CCN@*E. coli* containing medium was collected and stored at −80 °C for further analyzing. ^15^N-NMR measurements of ^15^NO_2_^–^ generation were made according to the method of Loscalzo et al.^36^. The ^15^N-NMR spectra were recorded with a Bruker 800-MHz Advance III spectrometer (Billerica, Biospin). Spectra were recorded at a frequency of 50.68 MHz and ^15^N-NMR data were collected with a sequence of 10-s relaxation delay and 300 pulse width. Data were managed with Fourier transformation. The chemical shift of ^15^N-pyridine in neutral media was defined as 0 ppm (to convert to the CH_3_NO_2_ scale, subtract ~75 ppm).

### Luminol luminescence assay

The luminol luminescence assay was performed according to the method of Zweier et al.^37^. The luminescence measurement of ONOO− generation was performed by using LB medium cultured CCN@*E. coli* (10^9^ CFU mL^−1^). Then, CCN@*E. coli* was irradiated under light irradiation ( > 630 nm, 30 mW cm^−2^, 15 min). Immediately after the irradiation, luminol (5-amino-2, 3-dihydro-1, 4-phthalazinedione, 500 mM) aqueous solution was mixed with CCN@*E. coli* containing LB medium (1:1, v:v) at 37 °C. The luminescence of the mixture was measured with IVIS spectrum.

### NAD^+^/NADH assay

NAD^+^/NADH levels were measured with Amplite™ NAD^+^/NADH assay kit. CCN@*E. coli* (10^9^ CFU mL^−1^ in LB medium) was irradiated under light irradiation ( > 630 nm, 30 mW cm^−2^, 15 min). Two hours after the irradiation, the CCN@*E. coli* containing medium was collected. Then CCN@*E. coli* was harvested by centrifugation (590 × *g*, 5 min) at 4 °C and resuspended with 0.8 mL lysis buffer for 15 min. The lysate was then centrifuged at 150 × *g* for 5 min at 4 °C. Fifty microliters of NAD/NADH reaction mixture was added into each well of NADH standard, blank control, and test samples to make the total NAD/NADH assay volume of 100 µL per well. The absorbance at 460 nm was monitored by UV−Vis spectrophotometer (Lambda Bio 40, PerkinElmer, USA).

### Cell viability assay

The MTT assay was used to measure the cytotoxicity of CCN@*E. coli*. 4T1 cells were seeded under the down chamber of 24-well transwell® (Corning Costar, USA) filters with a density of 5 × 10^4^ cells per well. CCN@*E. coli* was placed on the up chamber of the assay at a density of 1 × 10^6^, 1 × 10^7^, and 1 × 10^8^ CFU mL^−1^ (100 μL each well). Four hours after the irradiation ( > 630 nm, 30 mW cm^−2^, 15 min), the up chamber was removed. Twenty-four hours later, MTT (50 μL, 5 mg mL^−1^) was added into each well and co-incubated for another 4 h. The cultured medium in each well was replaced with dimethylsulfoxide (DMSO) (750 μL). The absorbance at 570 nm was measured with SpectraMax i3x multi-mode detection platform. The relative cell viability was counted according to the following formula: cell viability (%) = (OD_570 sample_ − OD_570 blank_) / (OD_570 control_ – OD_570 blank_) × 100%.

### DAF-FM DA assay

4T1 cells were seeded in the inside chamber of 3D-printed culture device with a density of 50,000 cells per well and incubated in 1640 medium containing 10% FBS for 24 h. Then, 0.2 μL of DAF-FM DA (5 mM) was added and stained for 15 min. CCN@*E. coli* (10^8^ CFU mL^−1^, 100 μL) was added in the outside chamber of the device. Remarkably after the irradiation ( > 630 nm, 30 mW cm^−2^, 15 min), CCN@*E. coli* and 4T1 cells were observed with Perkinelmer UltraView VoX 3D live cell imaging system.

### Dendritic cell maturation

Dendritic cell (DC) was differentiated from bone mesenchymal stem cell^34^. DC maturation was tested by co-culturing with supernatant of CCN@*E. coli + *light, CCN@*E. coli + *dark, *E. coli* and PBS treated 4T1 cell. The DC suspension was passed through a 40 μm nylon cloth to produce a single cell suspension. Cells were then stained with antibodies against CD11c (Biolegend, 1:800), CD80 (Biolegend, 1:500), and CD86 (Biolegend, 1:200). Flow cytometry analysis was performed on a BD Accuri C6.

### In vivo tumor targeting

All in vivo experiments were implemented in accordance with guidelines for laboratory animals established by the Wuhan University Center for Animal Experiment/A3-Lab. The in vivo fluorescence imaging of CCN@*E. coli* was performed on 4T1 tumor-bearing Balb/c mice. Two-hundred microliters of DIR labeled CCN@*E. coli* (10^8^ CFU mL^−1^) was i.v. injected into mice. Then, the in vivo accumulation of CCN@*E. coli* within mice was observed at different time points. For the 3D fluorescence image, transillumination fluorescence model was performed. Forty-eight hours after the injection, mice were sacrificed, and each organ was collected for ex vivo fluorescence imaging.

### Tumor permeating study

Balb/c mice (female, 9–10-weeks-old) bearing 4T1 tumors were sacrificed to collect the whole tumors when the tumors grew to ~9.0 mm. Tumors (*n* = 3 for each group) were cultured with DIR labeled CCN@*E. coli* or *E. coli* at a concentration of 10^8^ CFU mL^−1^ for 24 h. The fluorescence intensity of the whole tumor was observed with IVIS spectrum. Fluorescent images of tumor sections were observed with an inverted fluorescence microscope. For in vivo tumor permeating study, 4T1 tumor-bearing mice with a tumor diameter of ~9.0 mm were used. Two-hundred microliters of DIR labeled CCN@*E. coli* (10^8^ CFU mL^−1^) was i.v. injected. Two days after the injection, mice were sacrificed, and their tumors were collected for further whole tumor or tumor sections fluorescence imaging.

### CLARITY technique for tissue clearing

Two-hundred microliters CCN@*E. coli* (10^8^ CFU mL^−1^) was i.v. injected into 4T1 tumor-bearing mice. The CLARITY method was performed according to the previous report of Treweek et al.^38^. Briefly, mice were anesthetized and sacrificed 24 h after the irradiation, and then perfused with ice-cold PBS containing 0.5% (wt/vol) NaNO_2_ and 10 U mL^−1^ heparin. Mice tumors were collected and fixed with 4% paraformaldehyde at 37 °C for 2 h. To allow the sufficient penetration of monomer and initiator, tumor samples were then immersed in ice-cold PBS containing acrylamide monomer (4% wt/vol) and 2,2′-azobis [2-(imidazolin-2-yl)propane] dihydrochloride thermoinitiator (0.25% (wt/vol)) at 4 °C for 1 day. Then, the polymerization of polyacrylamide hydrogel was initiated by keeping this system in 37 °C water for 3 h with N_2_ protection. The as-prepared tumor/hydrogel hybrid was washed with 10% SDS and 0.01% NaN_3_ contained PBS on a water baths shaker at 37 °C for 4 days until the non-transparent tumor/hydrogel hybrid was turned into an optically transparent hydrogel. The CLARITY technique only removed lipid molecules among tissue, whereas biomacromolecule such as proteins or nucleic acids were retained. Thus, further immunofluorescence staining or TUNEL staining could be performed.

### In vivo bacteria clearance

Female Balb/c mice (5–6 weeks, *n* = 10) were used to study the bacteria clearance in vivo. Two-hundred microliters of CCN@*E. coli* (10^8^ CFU mL^−1^) was i.v. injected into mice. At different time points, mice were sacrificed, and the 16 S RNA (MG1655) level in liver, spleen, and kidney was measured. For antibiotics treated group, 100 μL of ampicillin (20 mg kg^-1^) was i.v. injected into mice.

### Mice body temperature and white blood cell count

Female Balb/c mice (5–6 weeks, *n* = 5) were used in this studied. CCN@*E. coli* (10^8^ CFU mL^−1^, 200 µL), lipopolysaccharide (250 μg mL^−1^, 200 µL) or PBS was i.v. injected into mice. At different time points, the body temperature of mice was measured with an electric thermometer (FR1DZ1, Microlife, Switzerland). WBC count of mice was measured with an auto hematology analyzer (MC-6200Vet, Icubio, China).

### Bioluminescence assay for monitoring NO generation

CT26 cells were stably transfected with the Nrf2 controlled luciferase expression plasmid using Lentivirus (Thermo Fisher Scientific) according to the manufacturer’s instructions. Neomycin (80 mg mL^−1^) was added to select the transfected cells. After CCN@*E. coli* treatment, d-Luciferin (Invitrogen) was added (with a final concentration of 150 μg mL^−1^). A SpectraMax i3x multi-mode detection platform (Molecular Device) was used to determine its bioluminescence intensity. For the in vivo experiment, 200 µL CCN@*E. coli* (10^8^ CFU mL^−1^) was i.v. injected into stably transfected CT26 tumor-bearing female Balb/c mice. Twenty-four hours post-injection, light irradiation was performed ( > 630 nm, 30 mW cm^−2^, 15 min). The in vivo bioluminescence intensity was measured with a Perkin-Elmer living image IVIS spectrum. Fifteen minutes before the observation, 100 μL of d-luciferin (3 mg d-luciferin per mice) was i.p. injected into tumor-bearing mice.

### MRI imaging for monitoring in vivo NO generation

The relaxivity of (MGD)_2_-Fe(II)-NO was measured on a 7.0 T MRI (BioSpec 70/20USR). Two-hundred microliters CCN@*E. coli* (10^8^ CFU mL^−1^) was i.v. injected into 4T1 tumor-bearing female Balb/c mice (*n* = 3). Twenty-four hours post-injection, light irradiation was performed ( > 630 nm, 30 mW cm^−2^, 15 min). MRI imaging was used to visualize the intratumoral NO level remarkably after the irradiation.

### Imaging apoptotic response in vivo

The visualization of tumor apoptosis in vivo was performed by using an annexin V-Cy5.5 conjugate^29^. Two-hundred fifty micrograms NHS-Cy5.5 was added into 10 mL annexin V (Biolegend, 0.1 mg mL^−1^) aqueous solution. The reaction was kept at room temperature for 24 h. Then, the solution was dialyzed against water and lyophilized to obtain the annexin V-Cy5.5 conjugate. For in vivo experiment, 200 µL DIR labeled CCN@*E. coli* (10^8^ CFU mL^−1^) was i.v. injected into 4T1 tumor-bearing female Balb/c mice. Twenty-four hours post-injection, light irradiation was performed ( > 630 nm, 30 mW cm^−2^, 15 min). Twenty-four hours after the light irradiation, mice were i.v. injected with 1 nmol of Cy5.5-annexin V in 200 μl of PBS, and Cy5.5 fluorescence in tumor region was observed with a Perkin-Elmer living image IVIS spectrum.

The intratumoral NO concentration was measured with the Griess reagent. Two-hundred microliters DIR labeled CCN@*E. coli* (10^8^ CFU mL^−1^) was i.v. injected into 4T1 tumor-bearing female Balb/c mice. Twenty-four hours post-injection, light irradiation was performed ( > 630 nm, 30 mW cm^−2^, 15 min). Four hours after the irradiation, mice were sacrificed and their tumors were collected. One-hundred milligrams of tumor tissue was homogenized in 1 ml of PBS, and the homogenate was stored at −80 °C for further analysis. For each sample, 50 μL of homogenate was added into 96-well plate and mixed with 50 μL of A medium (0.045 M p-sulfanilic acid, 5 M acetic acid). Five minutes later, 50 μL of B medium (0.035 M α-Naphthylamine, 5 M acetic acid) was added. The absorbance at 540 nm was measured.

### In vivo anti-cancer therapy

Female Balb/c mice were subcutaneously injected with 4T1 cells or CT26 cells (1 × 10^6^ cells per mice). Once tumors reached an approximate size of 100 mm^3^, mice were randomly divided into four groups with six mice in each group. Then, mice were received i.v. injection with 200 μL of CCN@*E. coli* (with a *E. coli* concentration of 1 × 10^8^ CFU mL^−1^), *E. coli*, CCN and PBS on the first day. Twenty-four hours after the injection, tumors were illuminated for 15 min. Tumor size and mice weight were measured immediately before the injection. Tumor volume was defined as *V* = *W*^2^ × *L* × 0.5, where *W* and *L* are the shortest and longest diameters of tumors, respectively. Investigators performing tumor measurements were blinded to treatment groups. Detailed information of all animal experiments was listed in Supplementary Table 1.

### Dose-dependence anti-cancer effect of CCN@*E. coli*

Female Balb/c mice were subcutaneously injected with 4T1 cells (1 × 10^6^ cells per mice). Once tumors reached an approximate size of 100 mm^3^, mice were randomly divided into five groups with five mice in each group. Then, mice were received intratumoral injection with 200 μL of CCN@*E. coli* (with a *E. coli* concentration of 1 × 10^6^ CFU mL^−1^, 1 × 10^7^ CFU mL^−1^, 1 × 10^8^ CFU mL^−1^, and 1 × 10^9^ CFU mL^−1^) and PBS on the first day. Twenty-four hours after the injection, tumors were illuminated for 15 min. Tumor size and mice weight were measured every day. Investigators performing tumor measurements were blinded to treatment groups.

### Proteomics sample pretreatment

Once tumors reached an approximate size of 200 mm^3^, 4T1 tumor-bearing mice were randomly divided into two groups with three mice in each group. Then, mice were received intravenously injected of 200 μL of CCN@*E. coli* (with a *E. coli* concentration of 1 × 10^8^ CFU mL^−1^) and PBS on the first day. Forty-eight hours after the injection, mice were sacrificed and their tumors were collected. Then, collected tumors were weighed, and homogenized in 200 μL TEAB dissolution buffer. The homogenate was further broken by the ultrasonic cell disruptor for 15 min, and centrifuged at 12,840 × *g* for 20 min. The supernatant was deposited by adding 800 μL cold acetone containing 10 mM DTT and centrifuged at 12,840 × *g* for 20 min at 4 °C (Optima L-100XP, Beckmen). To break the disulfide bond of protein, the precipitate was collected and mixed with 800 μL cold acetone and heated to 56 °C. The mixture was subsequently centrifuged at 12,840 × *g* for 20 min at 4 °C and lyophilized to obtain the protein sample. The sample could be stored at −80 °C for further uses. Total protein concentration was measured using the Bradford method. For protein digestion, 2 μg trypsin was added and then incubated overnight at 37 °C. Then, equal volume of 0.1% FA was added for acidize and peptides were purified on Strata-X C18 pillar. The dried peptides power was redissolved with 20 μL 0.5 M TEAB for peptides labeling.

The iTRAQ labeling and fractionation was according to our previous method^39^. Briefly, samples were labeled with iTRAQ Reagent-8 plex Multiplex Kit (AB Sciex U.K. Limited). Next, the labeled samples were fractionated using high-performance liquid chromatography (HPLC) system (Thermo DINOEX Ultimate 3000 BioRS) using a Durashell C18, and 12 fractions were collected for further analysis. Liquid chromatography-electrospray ionization-tandem mass spectrometry (LC-ESI-MS)/MS analysis was conducted with an AB SCIEX nanoLC-MS/MS (Triple TOF 5600 plus) system. Samples were analyzed by using a 90-min gradient from 2–30% (buffer A 0.1% (*v/v*) formic acid, 5% (*v/v*) acetonitrile, buffer B 0.1% (*v/v*) formic acid, 95% (*v/v*) acetonitrile). MS1 spectra were collected in the range of 350–1500 *m*/z for 250 ms. The 20 most intense precursors with charge state 2–5 were selected for fragmentation, and MS2 spectra were collected in the range 50–2000 *m*/*z* for 100 ms; precursor ions were excluded from reselection for 15 s. Data of all differential proteins were listed in Supplementary Data 1.

ProteinPilot Software v4.5 was used for analyzing original data. For protein identification, the Paragon algorithm, which was integrated into ProteinPilot was employed against Uniprot Mus musculus (86109 items) for database searching. Proteins with at least one unique peptide and unused value more than 1.3 were collected for further analysis. For protein abundance ratios measured using iTRAQ after normalized, we took a 1.5-fold change and *P*-value <0.05 as the threshold to identify significant changes. All differential proteins were listed in Supplementary Data 1.

To determine the biological and functional properties of all the identified proteins, identified protein sequences were mapped with Gene Ontology Terms (http://geneontology.org/). For this, homology search was first performed for all the identified sequences with a localized NCBI blast program against NCBI animal database. The *e*-value was set to <1 × 10^-5^, and the best hit for each query sequence was taken account for GO term matching. The GO term matching was performed with blast2go v4.5 pipeline5. Clusters of Orthologous Groups of Proteins System (COG, http://www.ncbi.nlm.nih.gov/COG/) was employed for the functional annotation of genes from new genomes and for research into genome evolution. To identify candidate biomarkers, we employed hypergeometric test to perform GO enrichment and KEGG pathway enrichment. To point out the protein–protein interactions, Search Tool for the Retrieval of Interacting Genes/Proteins (STRING) was employed (http://www.string-db.org/). All other pictures were drawn with R language (http://www.r-project.org/).

### Dead-cell proximity and Euclidean distance matrix analysis

The dead-cell proximity index was the ratio between the distance of CD86-positive DCs to caspase-3-negative cancer cells and the distance of CD86-positive DCs to caspase-3-negative cancer cells. The average dead-cell proximity index was measured from 25 independent areas. Dead-cell proximity indexes of each area were displayed in a box plot. Euclidean distance matrix analysis was used to compare the spatial distribution correlation between CD86 fluorescence and caspase-3 fluorescence. Then, Eq. 1 was used to calculate the mean distance between CD86-positive DCs and caspase-3-positive cancer cells.

1 $$d = \left[ {\sum \left( {f_{\rm r} - f_{\rm g}} \right)^2} \right]^{1/2}$$

Here, *d* is the Euclidean distance, *f*_r_ is the fluorescence intensity of red channel and *f*_g_ is the fluorescence intensity of the green channel. For Euclidean distance matrix analysis, 15 images were measured in each group and the result was presented in both box plot and histogram.

### Statistical analysis

Unless indicated otherwise, ‘‘center values’’ was defined as mean, and the error bars in each figure represent S.D. of at least three independent experiments. For statistical analyses, two-tailed Student’s *t*-tests were performed using Microsoft Excel 2013. A *P*-value of <0.05 was considered as statistically significant.

### Data availability

All relevant data are available from the authors.

## Electronic supplementary material

Supplementary Information

Descriptions of Additional Supplementary Files

Supplementary Data 1
