# Supplementary material for: Optically-controlled bacterial metabolite for cancer therapy
Source: Nat Commun. 2018 Apr 26;9:1680. doi: 10.1038/s41467-018-03233-9 (PMC5920064; doi:10.1038/s41467-018-03233-9)
Supplement: Supplementary file 1 — Supplementary Information [file 41467_2018_3233_MOESM1_ESM.docx]

**Optically-controlled Bacterial** **Metabolite for Cancer Therapy**

Zheng *et al*.


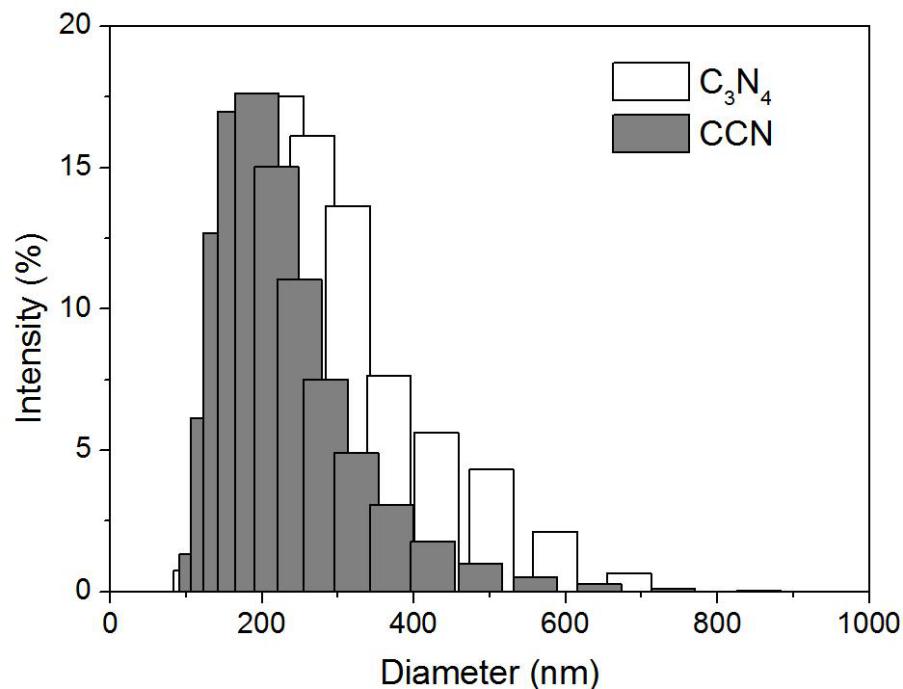


**Supplementary Figure 1 | Hydrodynamic sizes of C_3_N_4_ and CCN.** Dynamic light scattering (DLS) measurements of C_3_N_4_ and CCN in PBS (pH = 7.4).

**
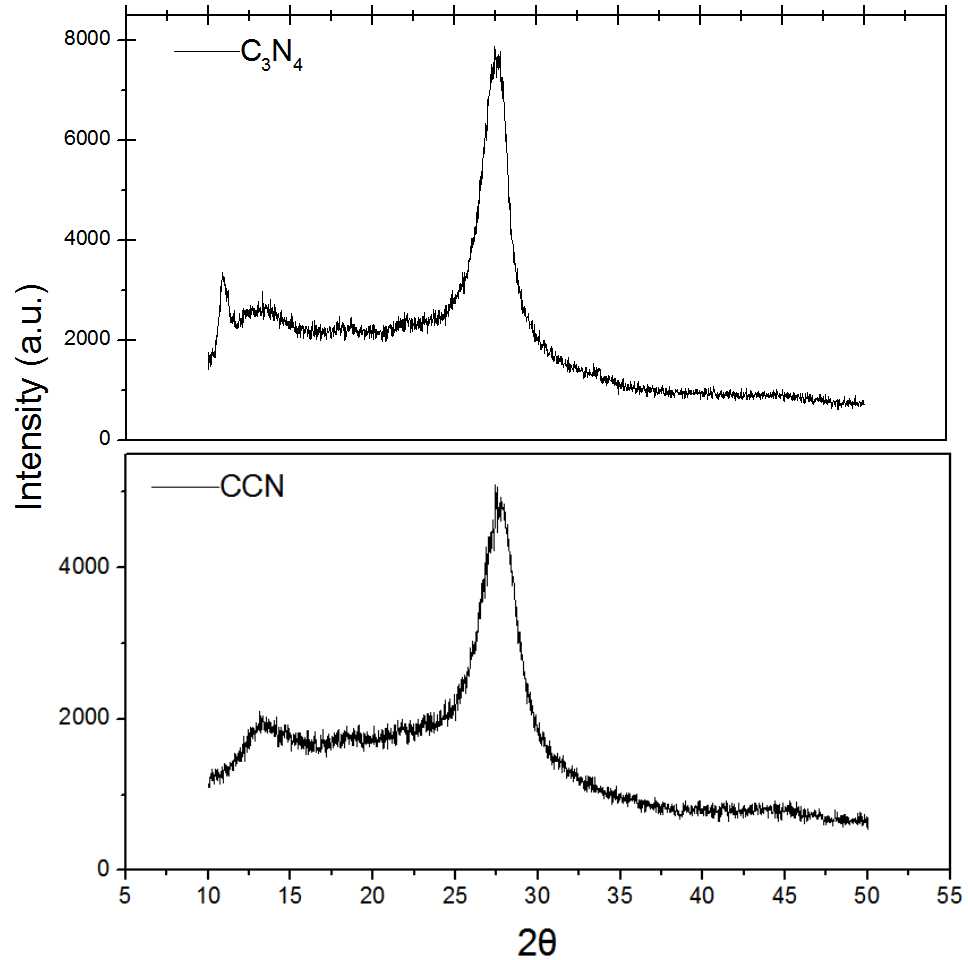
**

**Supplementary Figure 2 | XRD spectrum of C_3_N_4_ and CCN.** Revealing the successful synthesis of C_3_N_4_ and CCN.


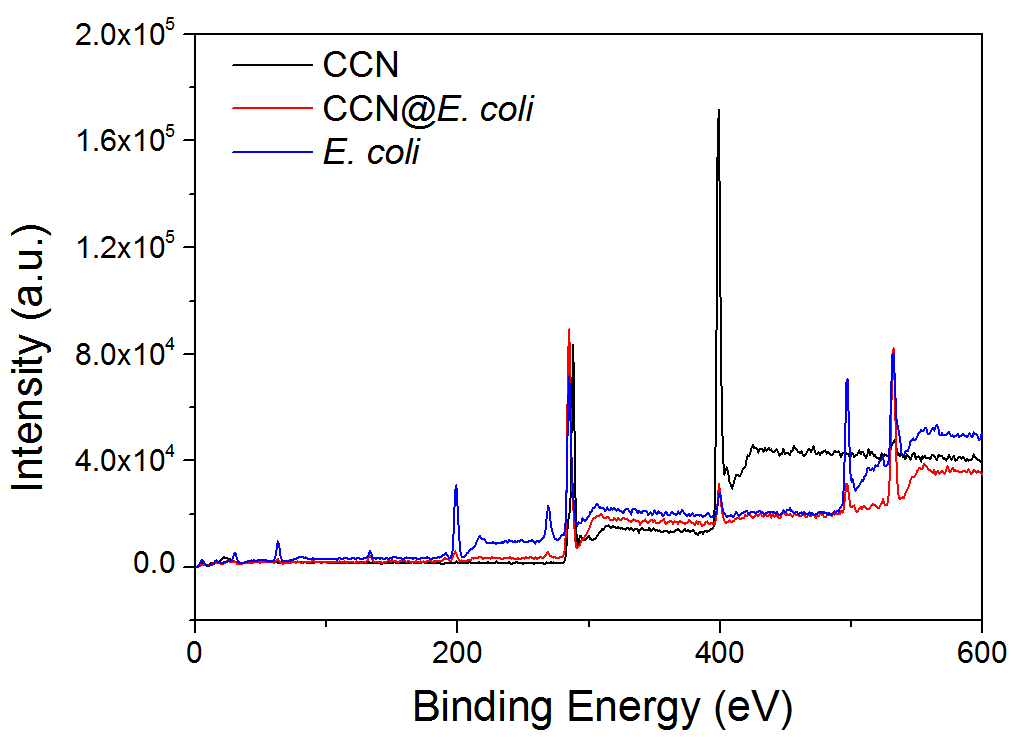


**Supplementary Figure 3 | XPS spectrum of C_3_N_4_, CCN, *E. coli* and CCN@*E. coli*.** The result indicating the successful surface modification of *E. coli* with CCN.


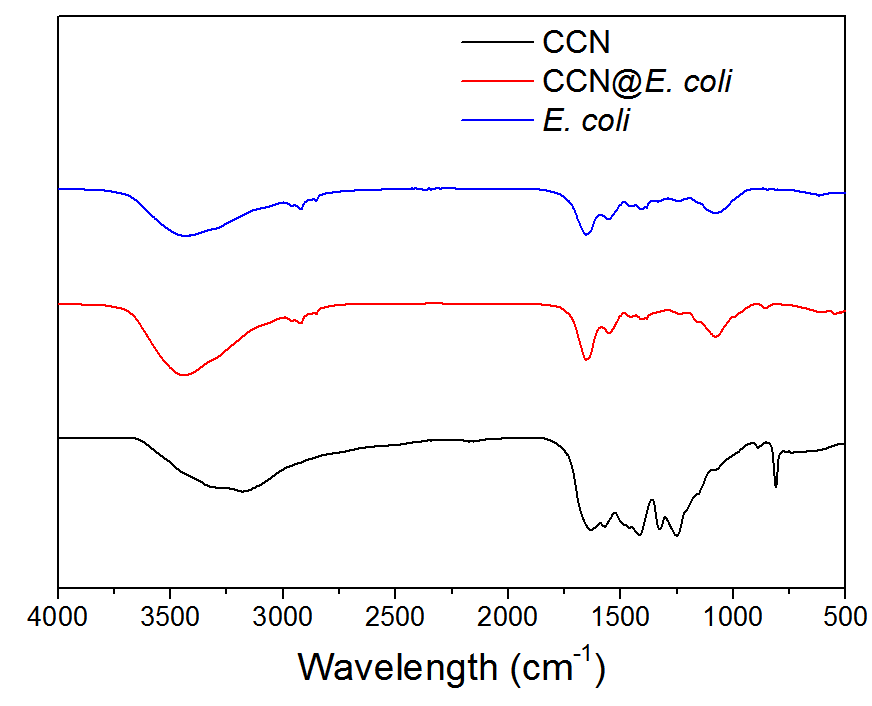


**Supplementary Figure 4 | FT-IR spectrum of CCN, *E. coli* and CCN@*E. coli*.**


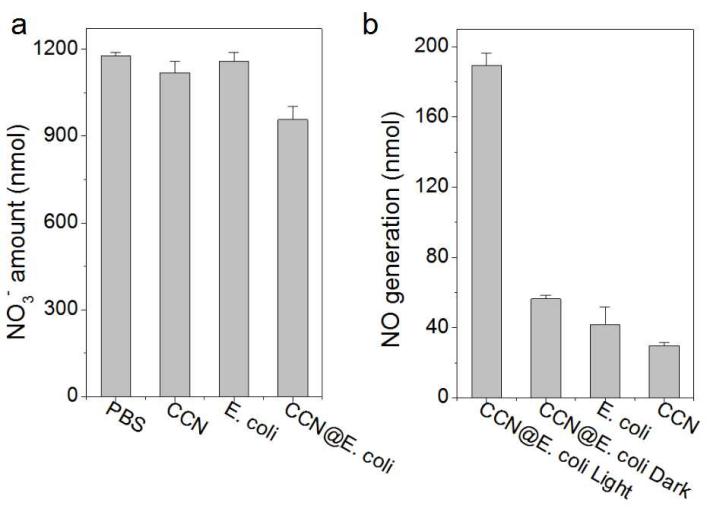


**Supplementary Figure 5 | *In vitro* study of PMT system. a** Nitrate reduction was detected by using ion chromatography for demonstrating the nitrite consumption of CCN@*E. coli* (10^9^ CFU, 2 mL) upon light irradiation. With the time prolonging, a continuous dropping of NO_3_^-^ content was observed. This result demonstrated that the reduced NO was indeed transformed from nitrate in the medium. **b** UV oxy-hemoglobin spectrophotometry for qualitatively demonstrating the generation of NO from CCN@*E. coli* (10^9^ CFU, 2 mL). The mean values and S.D. are presented.
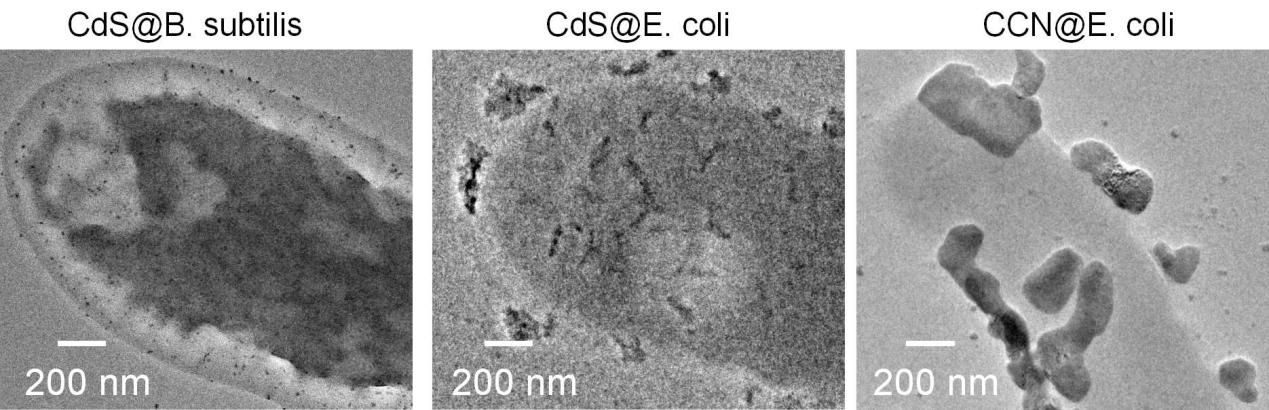


**Supplementary Figure 6 | TEM images of** **CdS@*B. subtilis*, CdS@*E. coli* and CCN @*E. coli*.**


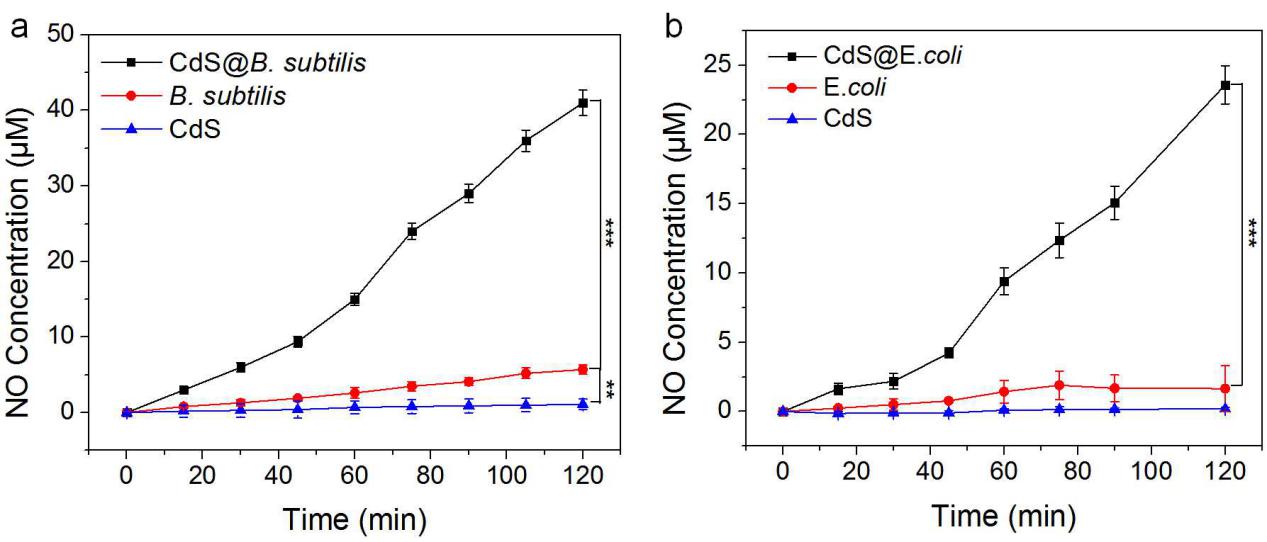


**Supplementary Figure 7 | NO generation ability of CdS-bacteria hybrid. a** NO generation of CdS@*B. subtilis*, *B. subtilis* and CdS. **b** NO generation of CdS@*E. coli*, *E. coli* and CdS. The mean values and error bars are defined as mean and S.D., respectively. Significance between every two groups was calculated using unpaired two-tailed Student's t test. **P* < 0.05, ***P* < 0.01, ****P* < 0.001.


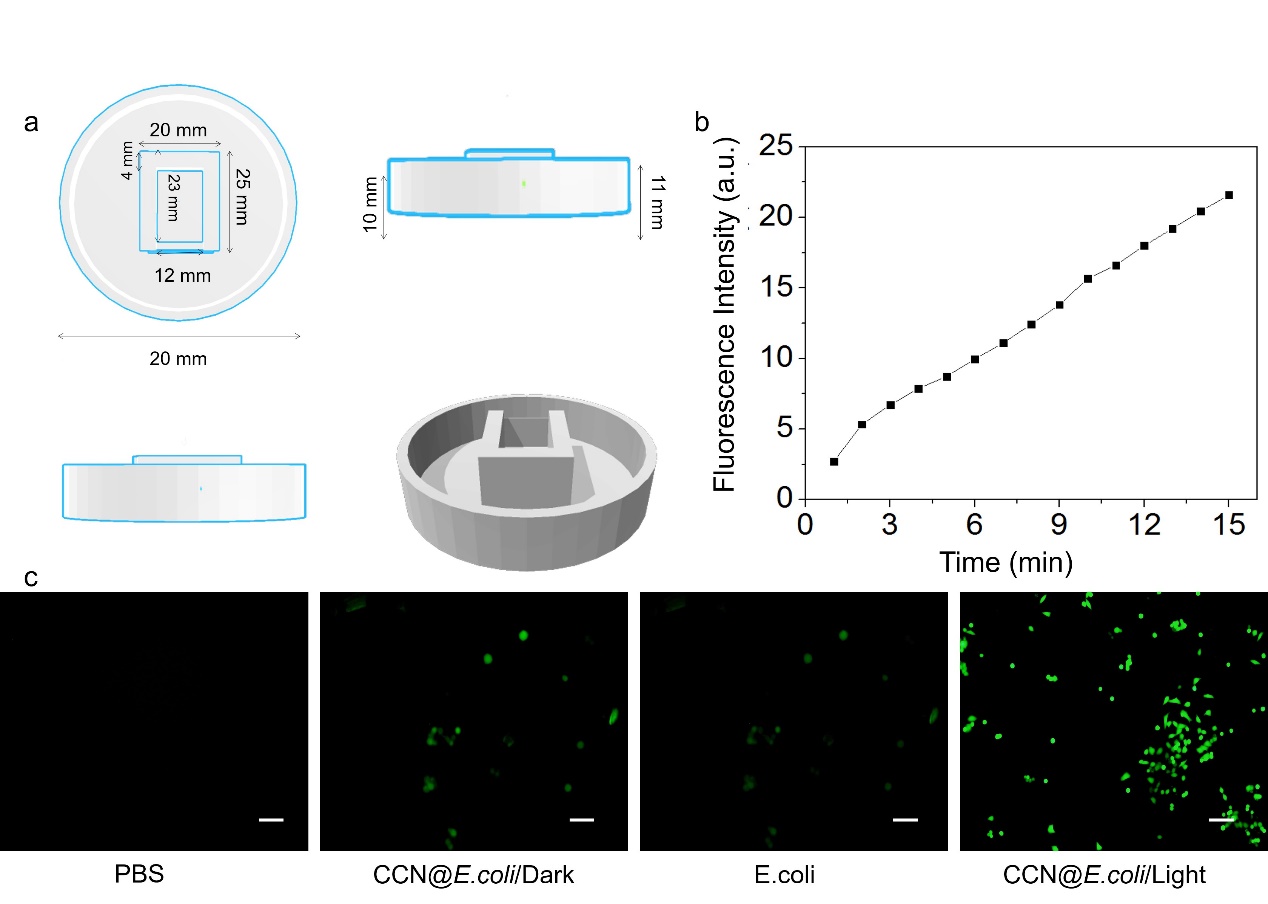


**Supplementary Figure 8 | *In vitro* non-contacting co-culture of bacteria with 4T1 cells. a** Three-view drawing of the 3D-printing co-culture system. **b** Fluorescence intensity-time curve of the NO generation in the co-culture system. **c** DCF-FM DA for visualize the intracellular NO generation after treated with PBS, CCN@*E. coli*/dark, *E. coli* and CCN@*E. coli*/light (Scale bar: 100 μm).


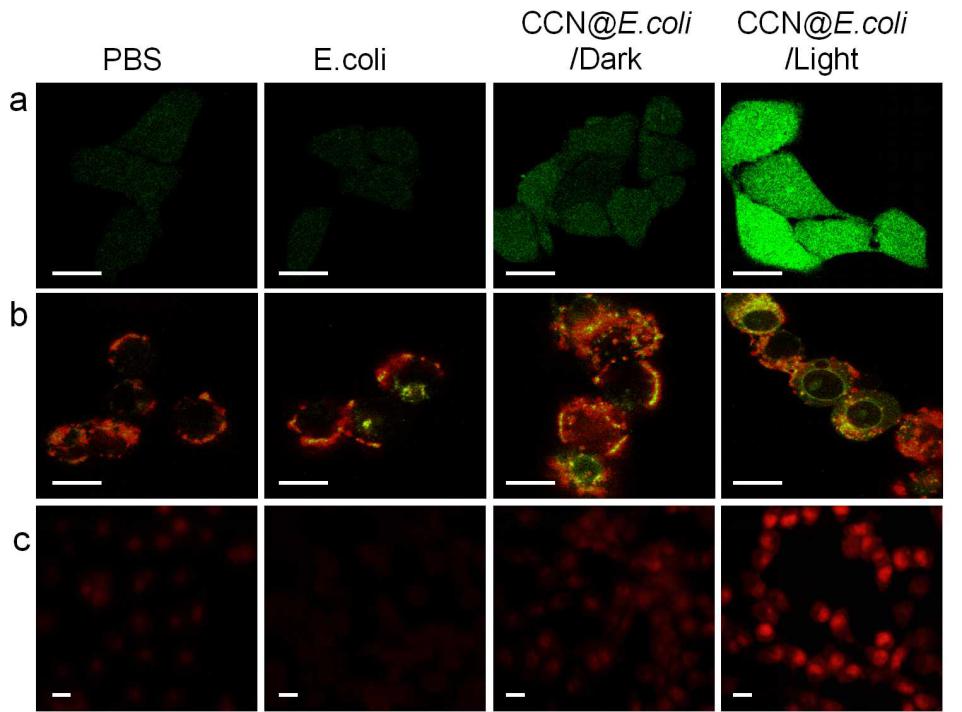


**Supplementary Figure 9 | Fluorescence imaging of intracellular oxidative stress level. a** DCFH-DA assay for detecting intracellular ROS generation. **b** JC-1 assay for illustrating mitochondrial membrane potential loss. **c** Dihydroethidium staining for measuring the superoxide generation (Scale bar: 20 μm).

**
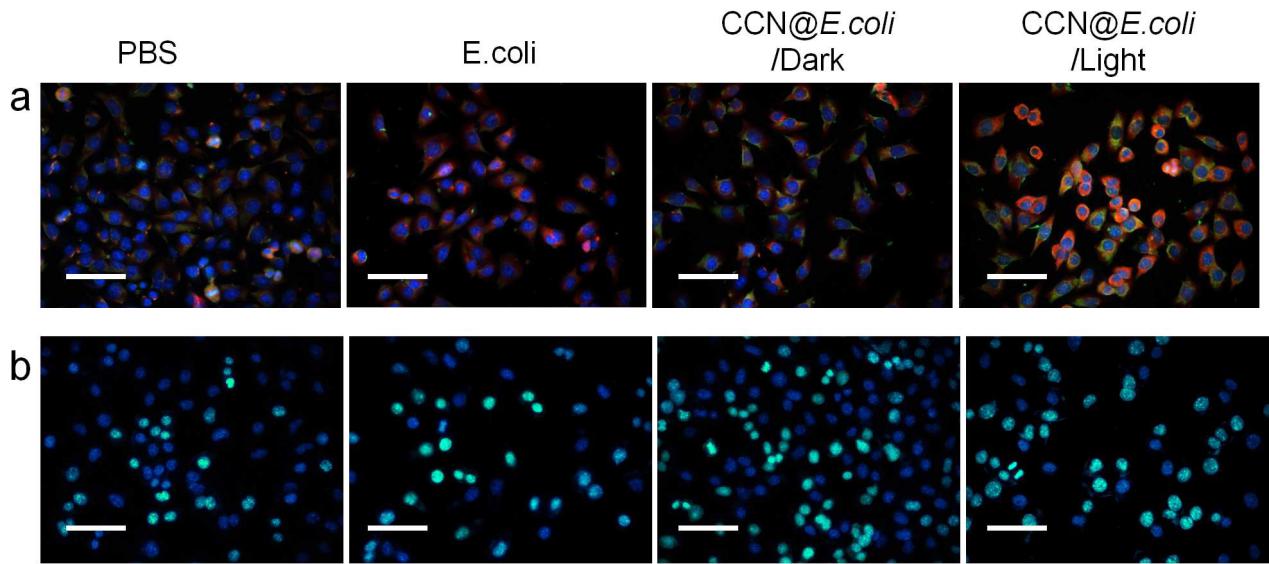
**

**Supplementary Figure 10 | Fluorescence imaging of cell apoptosis.** **a** Immunofluorescent staining of Bax and active-caspase-3 of PBS, *E. coli*, CCN@*E. coli*/dark and CCN@*E. coli*/light treated 4T1 cells. **b** TUNEL staining images of PBS, *E. coli*, CCN@*E. coli*/dark and CCN@*E. coli*/light treated 4T1 cells (Scale bar: 100 μm).


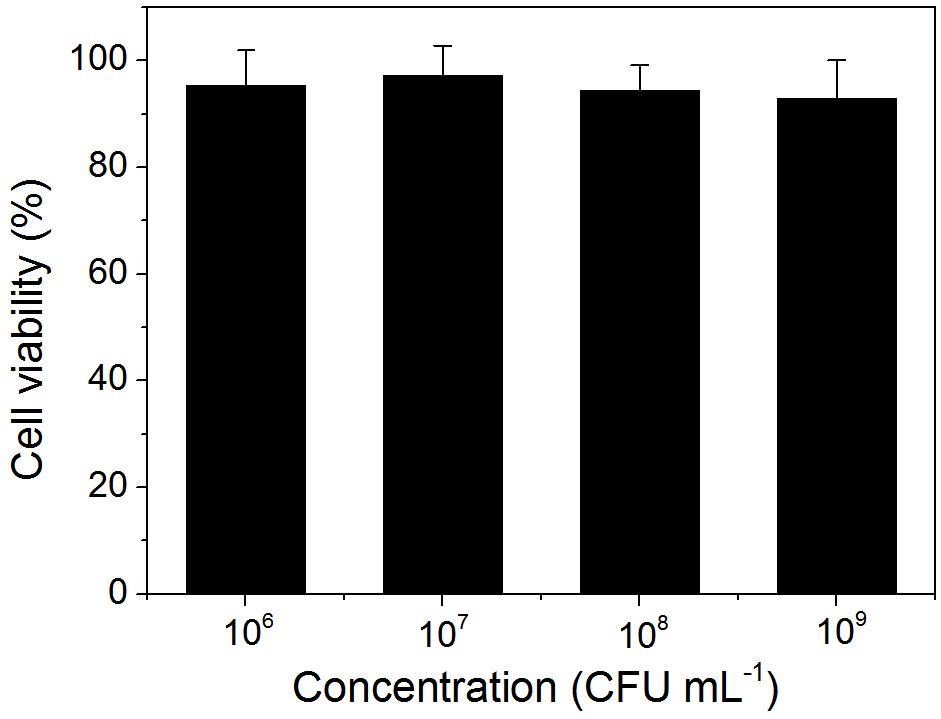


**Supplementary Figure 11 | Cell viability assay of 4T1 cells after co-incubation with CCN@*E. coli* in the dark condition.** The mean values and S.D. are presented.


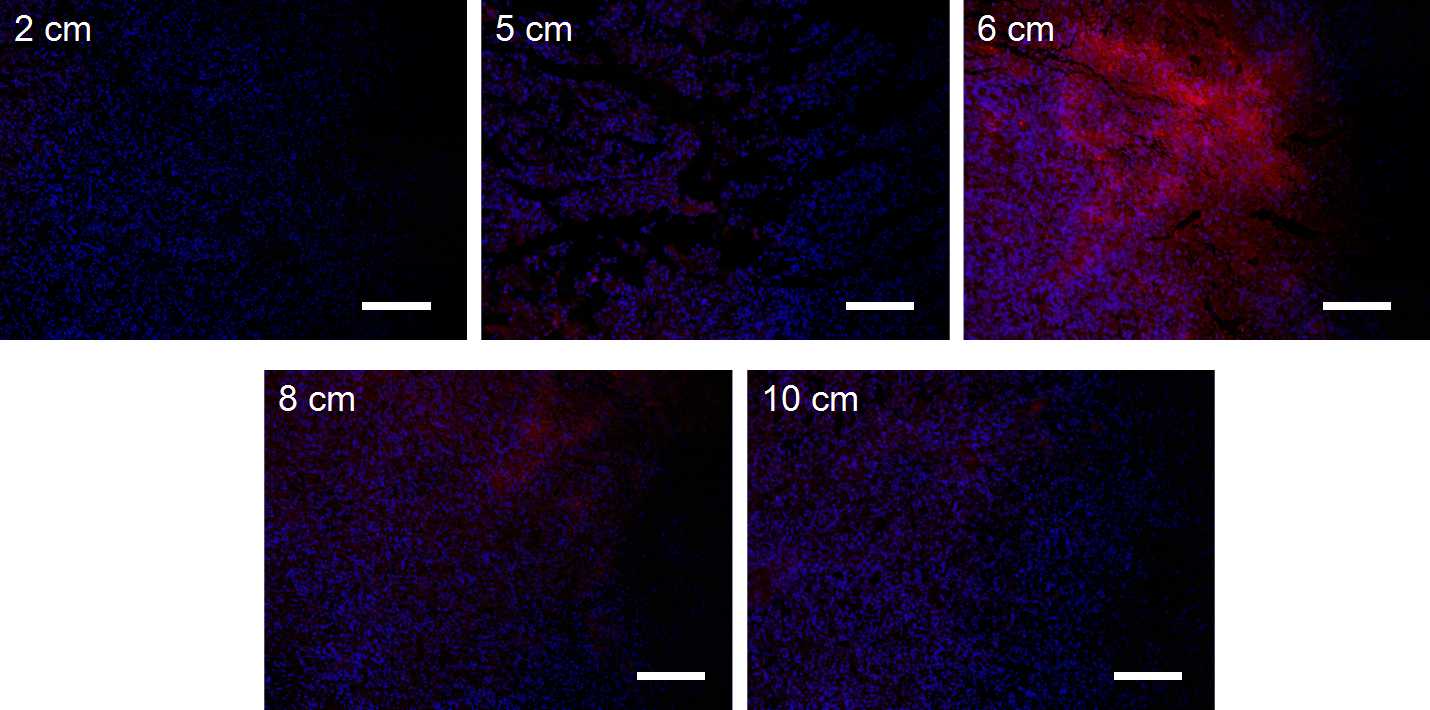


**Supplementary Figure 12 | Transverse tumour sections of CCN@*E. coli* after targeting.** Tumor sections were acquired at different tumor depths (Scale bar: 200 μm).

**
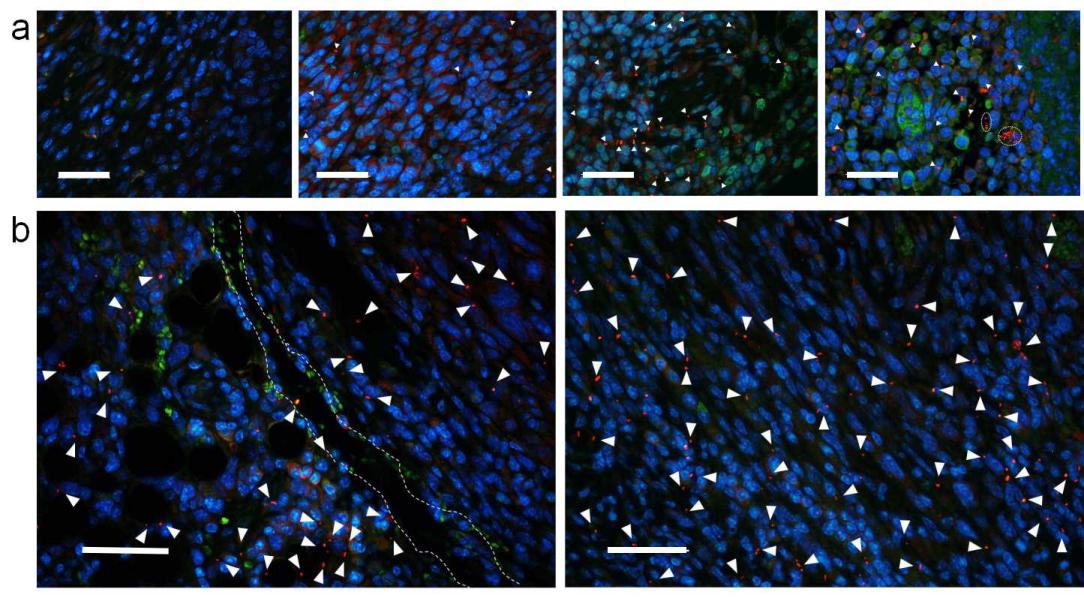
**

**Supplementary Figure 13 | 2D** **immunofluorescent imaging of** **CCN@*E. coli*, tumor hypoxic region and tumor vascular.** **a** Immunofluorescent imaging for estimating distributions of intratumoral CCN@*E. coli* and tumor hypoxic region (Blue: DAPI; Green: carbonic anhydrase 9; Red: *E. coli*; Arrows indicated the *E. coli* location). **b** Immunofluorescent imaging for estimating distributions of intratumoral CCN@*E. coli* and tumor vascular (Blue: DAPI; Green: CD31; carbonic anhydrase 9; Red: *E. coli*; Arrows indicated the *E. coli* location; Scale bar: 200 μm).

**
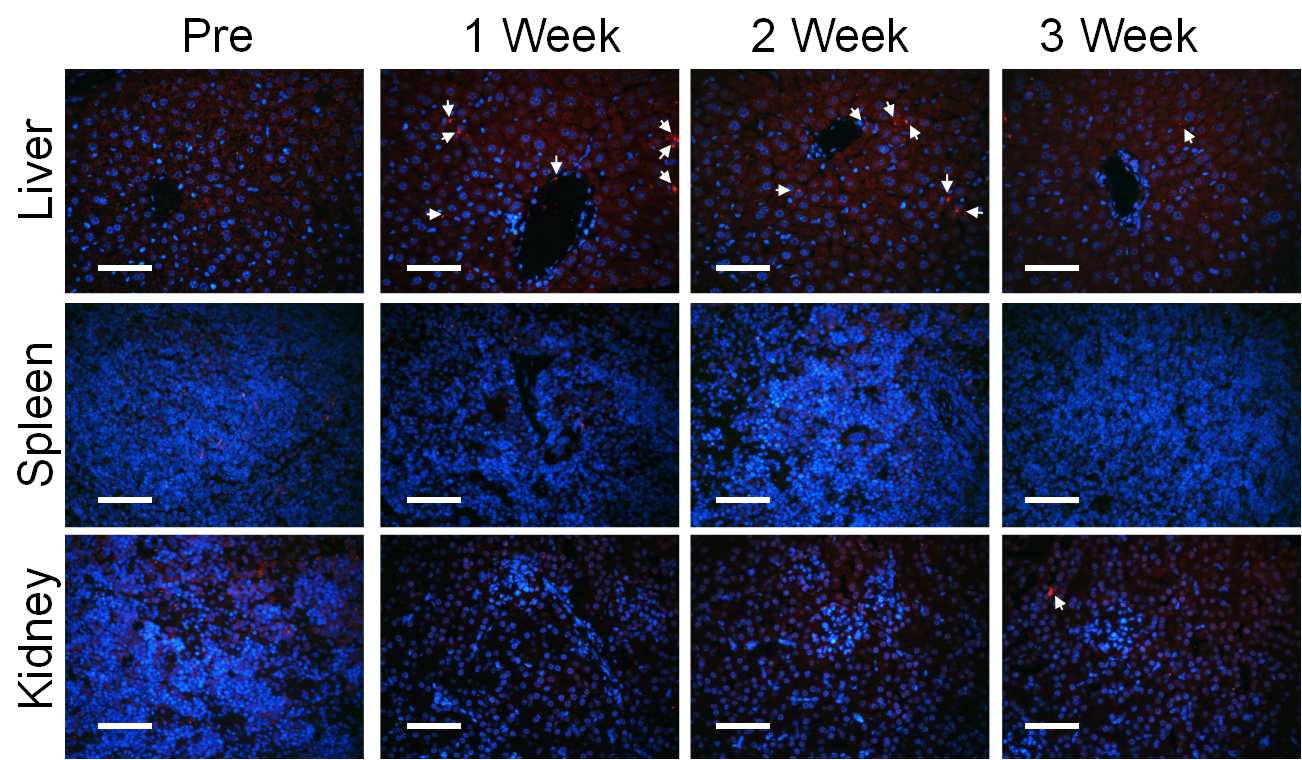
**

**Supplementary Figure 14 | Immunofluorescence images of CCN@*E. coli* within liver, spleen and kidney.** Arrows mark the residual *E. coli* within each organ (Scale bar: 200 μm).

**
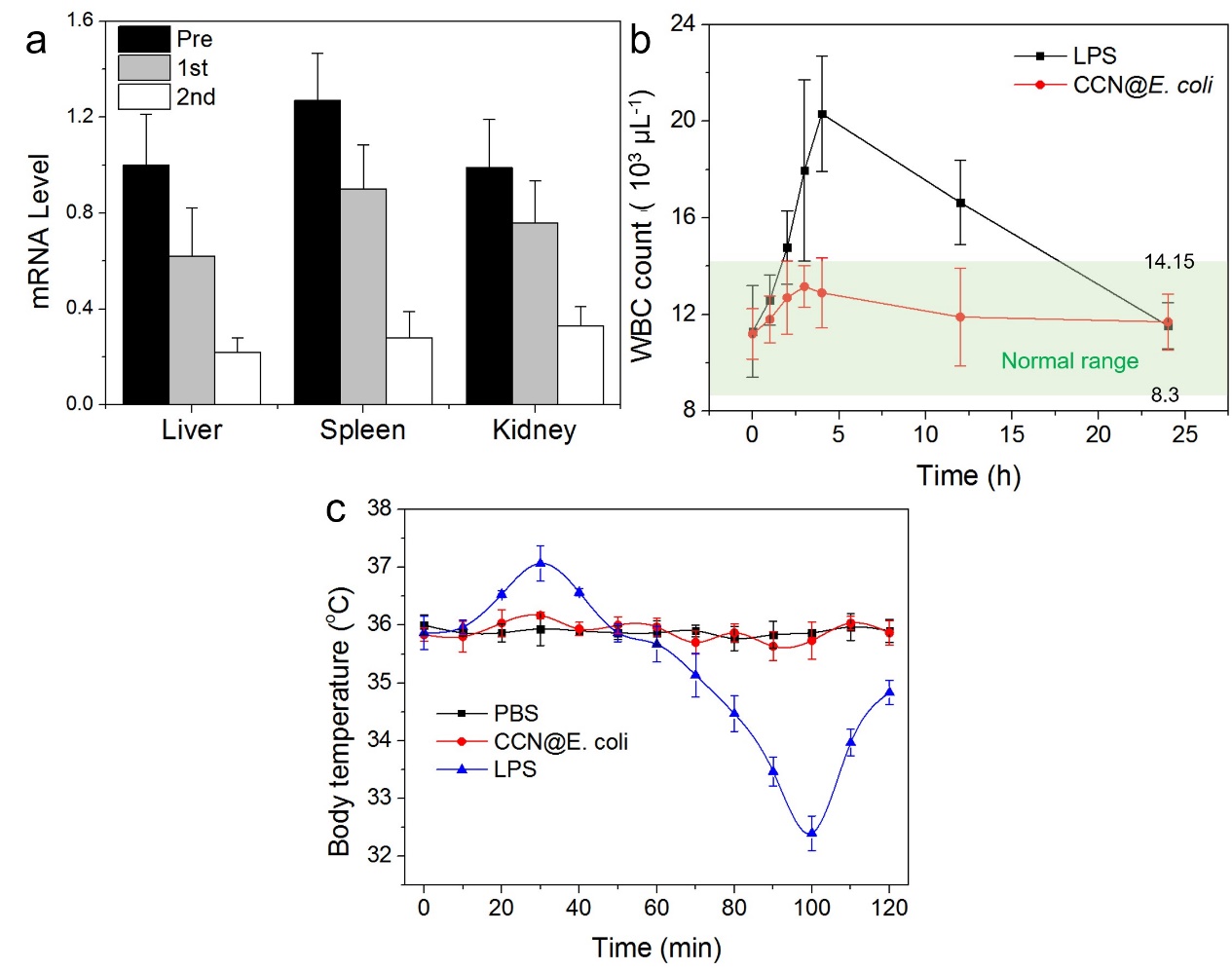
**

**Supplementary Figure 15 | *In vivo* bio-safety studies of CCN@*E. coli*. a** Controllable elimination of *CCN@E. coli*. PCR analysis of bacteria clearance after injection of Penicillin (0.6 mg kg^-1^). 16S RNA of *E. coli* MG1655 in liver, spleen and kidney were measured post injection. **b** WBC count after treatment with CCN@*E. coli.* at different time points. **c** Body temperature curve of female Balb/c mice after treatment with CCN@*E. coli.* The mean values and S.D. are presented.

**
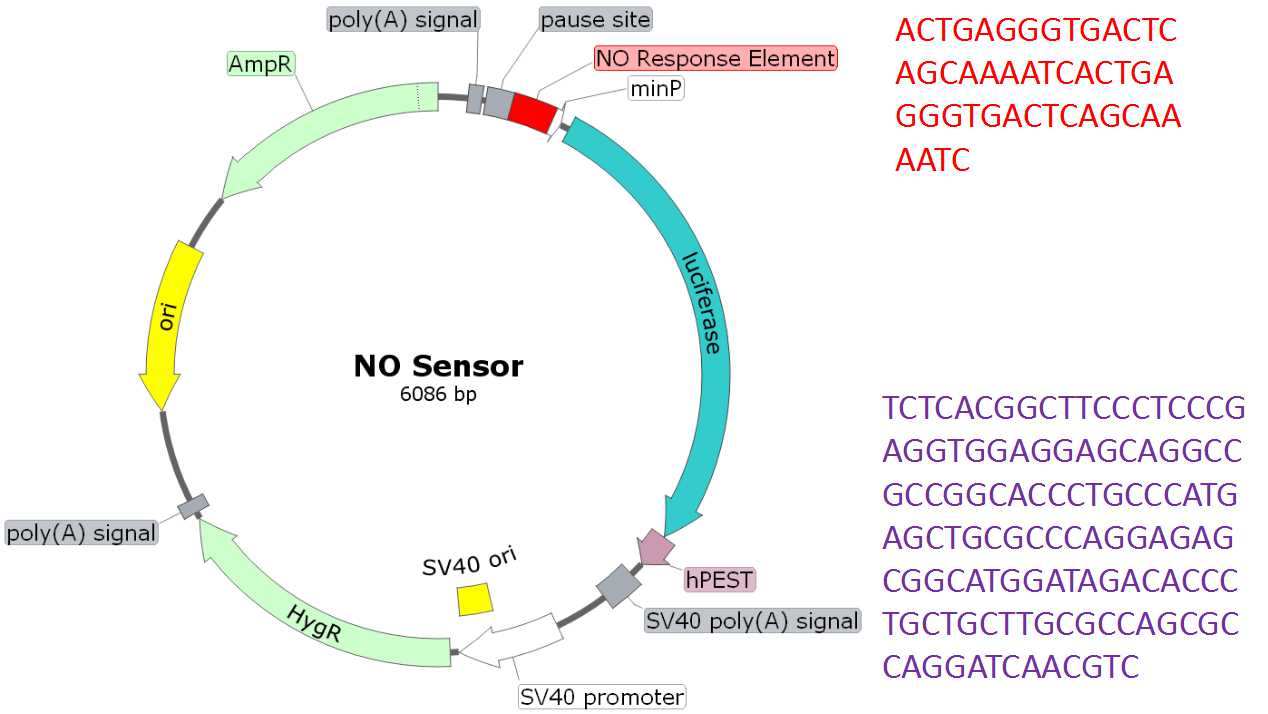
**

**Supplementary Figure 16 | Schematic illustration of the plasmid design.** Plasmid profile of the ROS sensor with oxidative stress responsive elements drived expression of fast-degraded luciferase proteins.

**
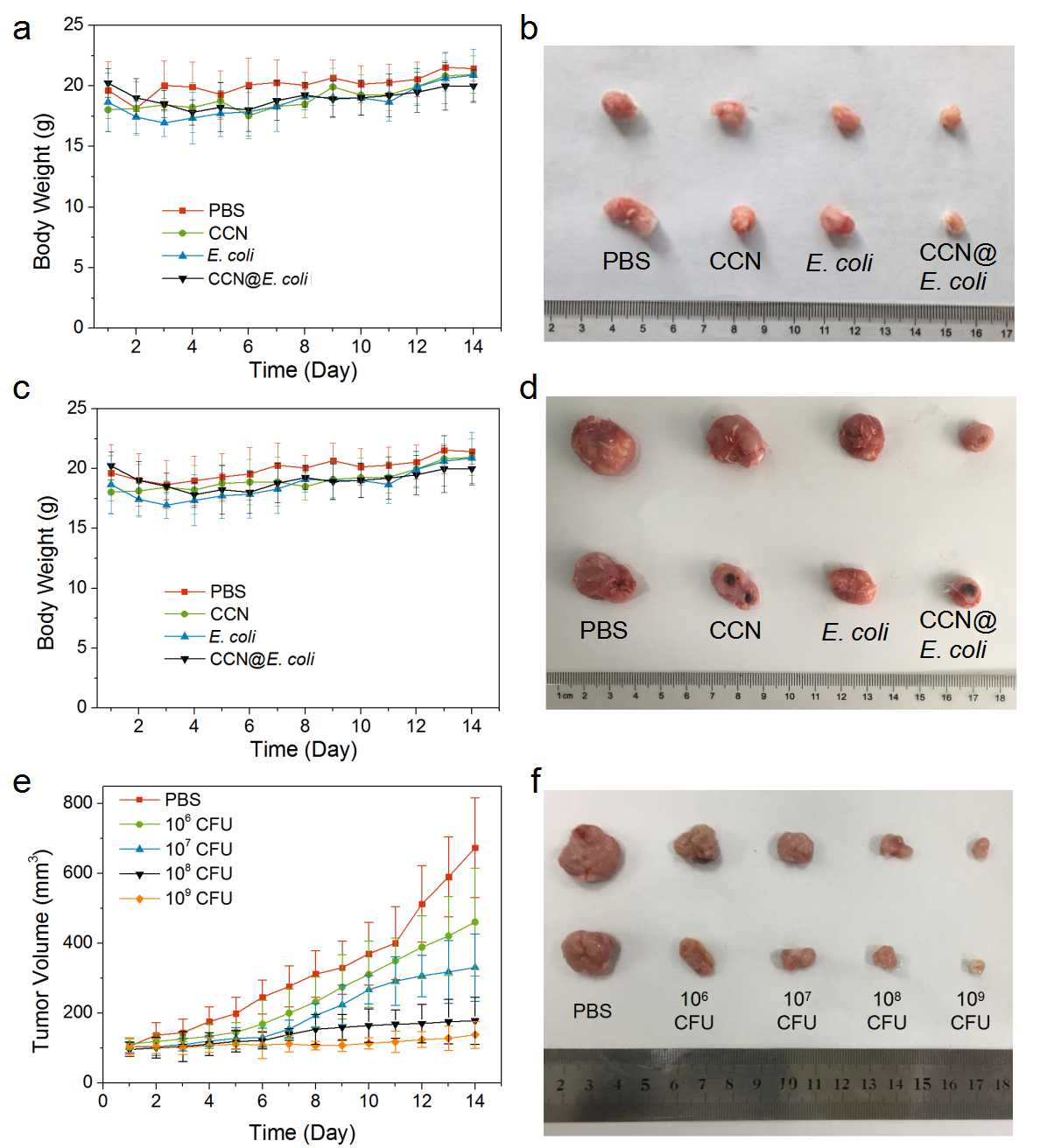
**

**Supplementary Figure 17 | *In vivo* anti-cancer study. a** Body weight curves of CT26 tumor bearing mice during treatment with PBS, CCN, *E. coli* and CCN@*E. coli*. **b** CT26 tumor image at the end of treatments. **c** Body weight curves of 4T1 tumor bearing mice during treatment with PBS, CCN, *E. coli* and CCN@*E. coli*. **d** 4T1 tumor image at the end of treatments. **e** Dose-dependent therapeutic effect of CCN@*E. coli* in 4T1 tumor bearing Balb/c mice. **f** 4T1 tumor image at the end of CCN@*E. coli* treatment with different doses. Data are mean ± s.d. (**a**, **c**, **e**).

**
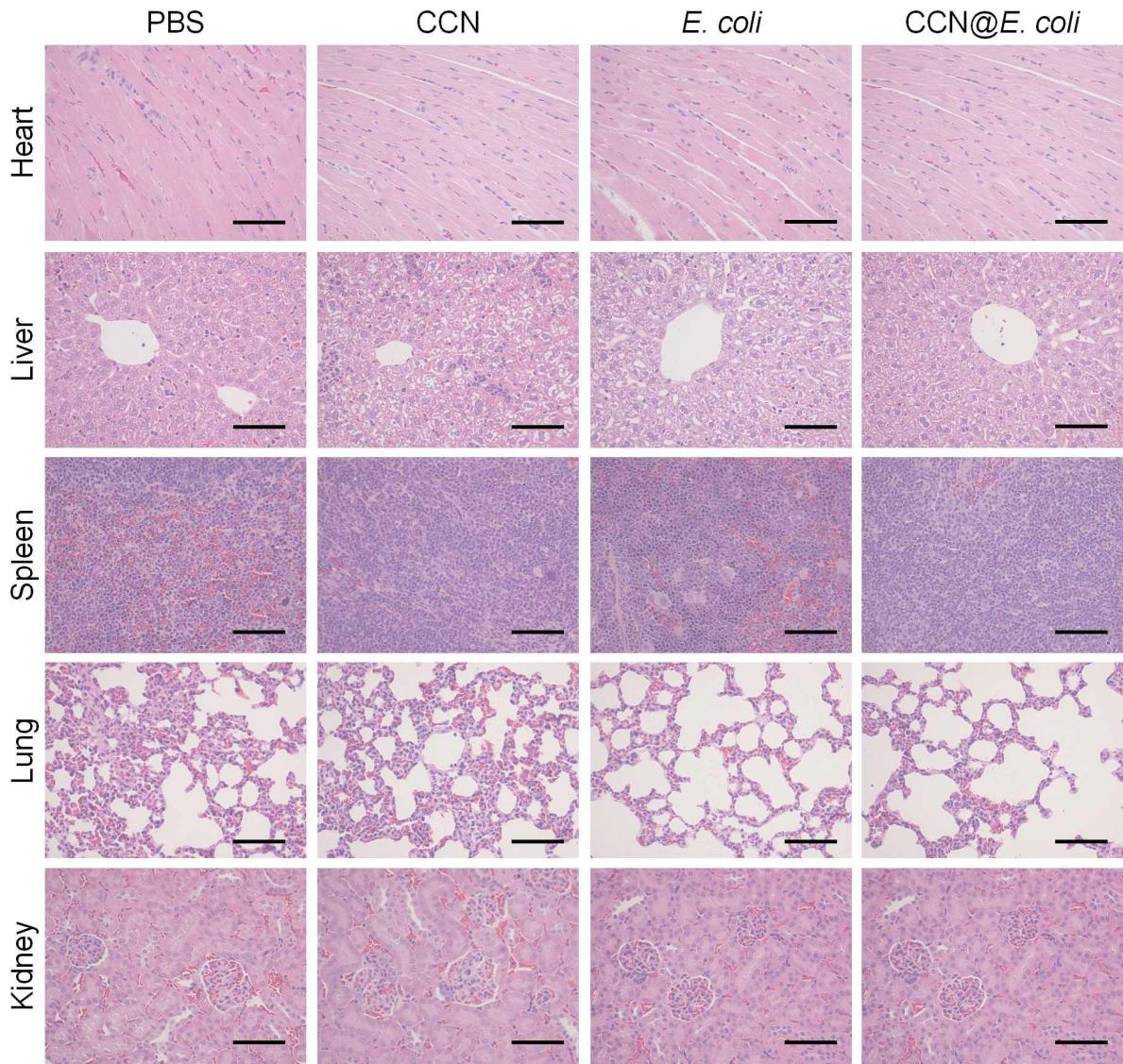
**

**Supplementary Figure 18 | H&E staining of materials treated mice tissue.** H&E staining of mice heart, liver, spleen, lung and kidney after 15 days of treatment with PBS, CCN, *E. coli* and CCN@*E. coli* (Scale bar: 100 μm).

**
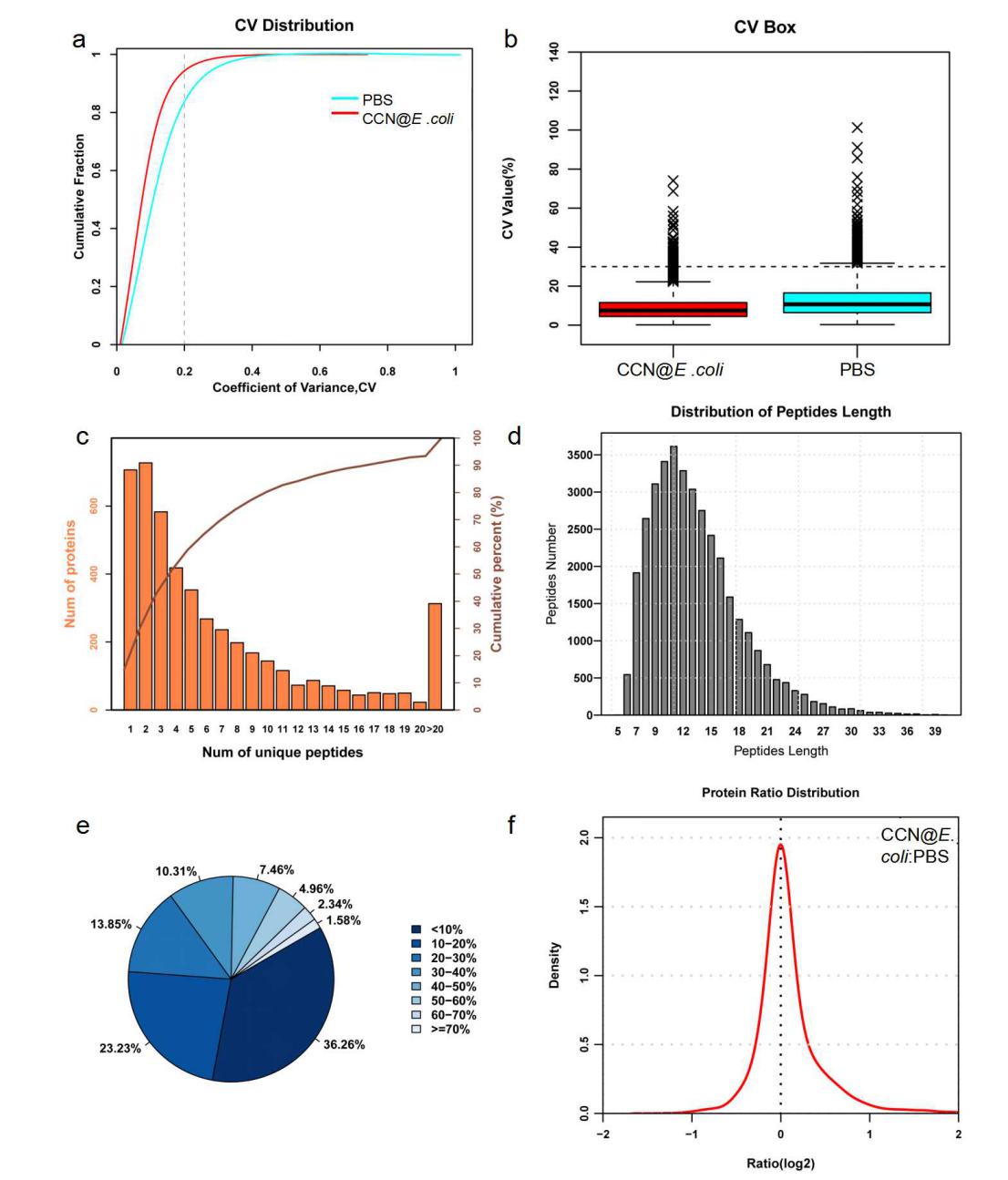
**

**Supplementary Figure 19 | Identifying differentially expressed proteins in PMT treated mice tumor.** **a** The CV distribution of the relative abundance of peptide features that were aligned in the three repeat LC-MS/MS analyses. **b** The CV box of the relative abundance of peptide features that were aligned in the three repeat LC-MS/MS analyses. **c** Overview of unique peptides distribution. **d** Overview of peptides length distribution. **e** Bar of pie chart of different percent of protein coverage (peptides with confidence≥95%). **f** Overview of protein abundance distribution between differential proteins. Data are median (**b**) linked with individual values.

**
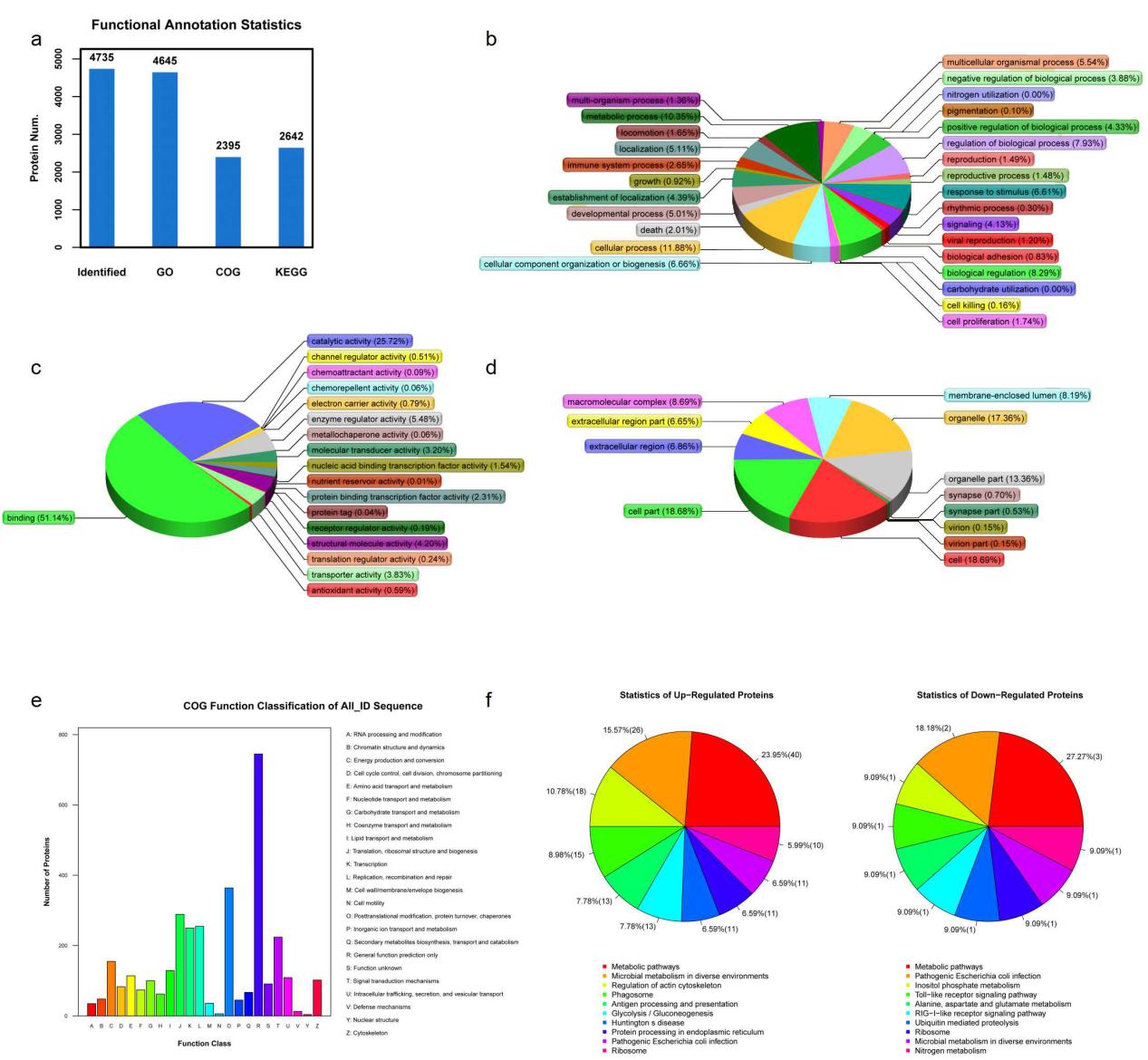
**

**Supplementary Figure 20 | Analysis of the proteomics data.** **a** The number of differentially expressed proteins analyzed by different functional annotation statistics. **b** Percent of proteins in each classification of biological process. **c** Percent of proteins in each classification of cellular component. **d** Percent of proteins in each classification of molecular function. **e** COG function classification of all identified proteins. **f** Statistics of KEGG pathways of up-regulated and down-regulated differential expressed proteins in PMT group as compared with PBS group.

**
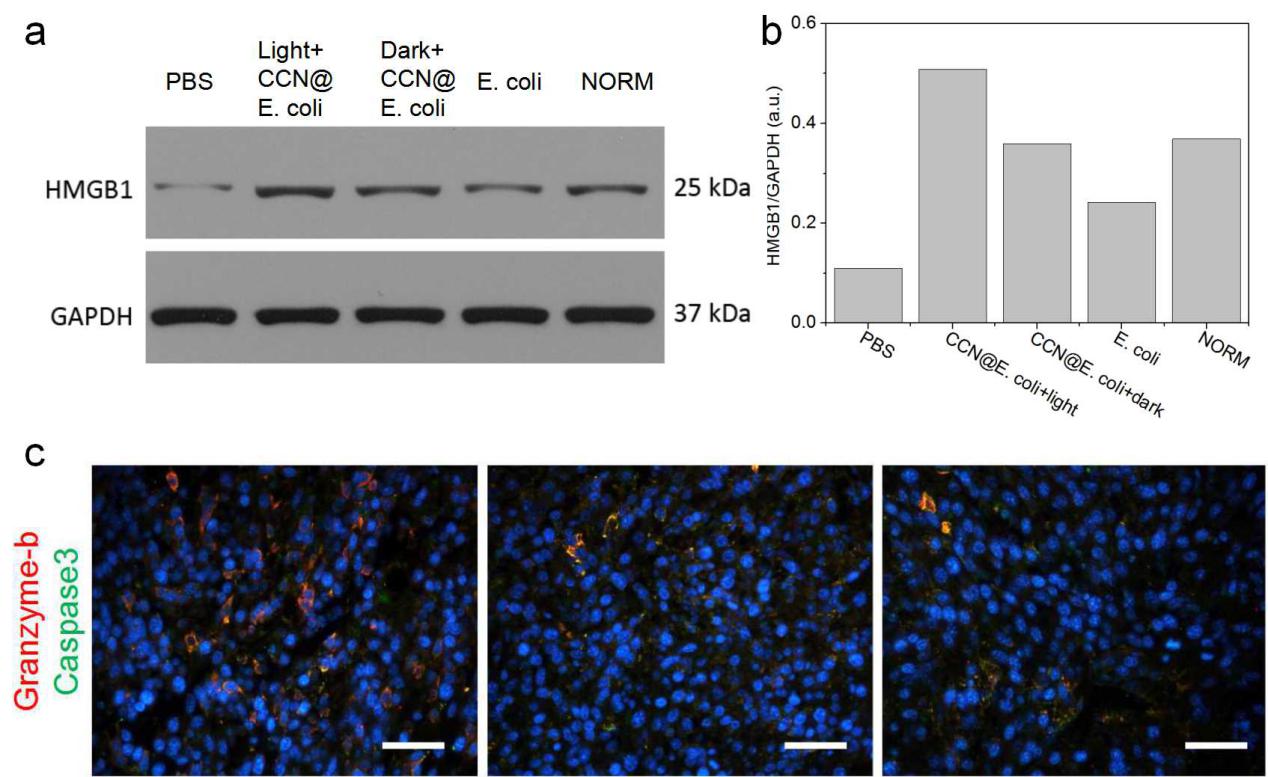
**

**Supplementary Figure 21 | Immunological effect of PMT treatment.** **a** Western blot assay for analyzing HMGB-1 level in DC cells. **b** Semi-quantitative analysis of HMGB-1 level in DC cells. **c** Immunofluorescent images for visualizing the colocalization between granzyme-b and caspase-3 (Scale: 100 μm).

**Supplementary Table 1 | Detailed information about animals experiments^*^**

| Experiment | Material | Dose  (per mice) | *n* | Method of administration | Method of irradiation |
| --- | --- | --- | --- | --- | --- |
| *In vivo* tumor targeting | DIR labeled CCN@*E. coli* | 10^8^ CFU mL^-1^  200 µL | 5 | *i.v.* | non |
| Tumor permeating study | DIR labeled CCN@*E. coli* | 10^8^ CFU mL^-1^  200 µL | 3 | *i.v.* | non |
| Tissue clearing | DIR labeled CCN@*E. coli* | 10^8^ CFU mL^-1^  200 µL | 3 | *i.v.* | 30 mW cm^-2^, 15 min |
| *In vivo* bacteria clearance | CCN@*E. coli* | 10^8^ CFU mL^-1^  200 µL | 10 | *i.v.* | non |
| Bioluminescence assay for monitoring NO generation | CCN@*E. coli* | 10^8^ CFU mL^-1^  200 µL | 3 | *i.v* | 30 mW cm^-2^, 15 min |
| MRI imaging for monitoring *in vivo* NO generation | CCN@*E. coli* | 10^8^ CFU mL^-1^  200 µL | 3 | *i.v* | 30 mW cm^-2^, 15 min |
| Imaging apoptotic response *in vivo* | DIR labeled CCN@*E. coli* | 10^8^ CFU mL^-1^  200 µL | 3 | *i.v* | 30 mW cm^-2^, 15 min |
| *In vivo* anti-cancer therapy  *In vivo* anti-cancer therapy  *In vivo* anti-cancer therapy | CCN@*E. coli* | 10^8^ CFU mL^-1^  200 µL | 6  6  6 | *i.v*  *i.v*  *i.v* | 30 mW cm^-2^, 15 min |
|  | *E. coli* | 10^8^ CFU mL^-1^  200 µL |  |  | 30 mW cm^-2^, 15 min |
|  | CCN | 5 mg mL^-1^  200 μL |  |  | 30 mW cm^-2^, 15 min |
| Proteomics sample pretreatment | CCN@*E. coli* | 10^8^ CFU mL^-1^  200 µL | 3 | *i.v* | 30 mW cm^-2^, 15 min |
| Dose-dependent anti- cancer therapy  Dose-dependent anti-cancer therapy  Dose-dependent anti- cancer therapy  Dose-dependent anti- cancer therapy | CCN@*E. coli*  CCN@*E. coli*  CCN@*E. coli*  CCN@*E. coli* | 10^9^ CFU mL^-1^  50 µL  10^8^ CFU mL^-1^  50 µL  10^7^ CFU mL^-1^  50 µL  10^6^ CFU mL^-1^  50 µL | 5  5  5  5 | *i.t*  *i.t*  *i.t.*  *i.t* | 30 mW cm^-2^, 15 min  30 mW cm^-2^, 15 min  30 mW cm^-2^, 15 min  30 mW cm^-2^, 15 min |

**^*^** All animal experiments were performed on 6-week-old female Balb/c mice.

**Supplementary Note 1:**

**XRD analysis.** Phase structure and properties of C_3_N_4_ and carbon-dot-decorated C_3_N_4_ (CCN) were investigated by using powder X-ray diffraction (XRD). Two basic diffraction peaks were found in pure C3N4 and CCN at 27.4° and 13.1°. The observed high intensity peak observed at 27.4° is referred to as (002) and the interlayer stacking distance is 0.326 nm which reflex graphitic like structures. Another peak observed at 13.1° indexed as (100). This peak corresponds to in-plane structural packing motif of a condensed tri-s-triazine unit, with an interplanar distance of d = 0.675 nm^1^. And after carbon-dot was decorated, just the peak at ca. 13.1° was decreased. The patterns reveal that the C_3_N_4_ atomic structure was largely retained.

**FT-IR analysis.** The formation of CCN, *E. coli* and CCN@*E. coli* has been clearly observed through FTIR spectroscopy. For CCN, the spectrum shows several peaks at 1200–1650 cm^−1^, which is related to the typical breathing modes of CN heterocycles. The characteristic peak at 805 cm^-1^ is related to the breathing mode of tri-*s*-triazine units. More importantly, the peak at 805 cm^-1^ also can be observed in CCN@ *E. coli,* while can’t be observed in *E. coli.* This observation confirmed the formation of CCN@*E. coli.*

**Supplementary Note 2:**

**TEM images of CdS@*B. subtilis*, CdS@*E. coli* and CCN @ *E. coli*.** The TEM images of CdS@*B. subtilis* CdS@*E. coli* and CCN@*E. coli* hybrid systems showed that high densities of nano-sized particles were formed on the cells (Supplementary Fig. 6). In CdS@*B.* *subtilis*, clusters of highly dispersed smaller nanoparticles with a diameter of around 10 nm was observed. However, on the surface of CdS@*E. coli*, aggregated CdS could be observed. Therefore, the nanosized particles that precipitate on the surface of bacteria might exhibit good photocatalytic activity.

**NO generation ability of CdS-bacteria hybrid.** To further test whether the photocatalytic material-bacteria hybrid could overcome the challenge of photo-controlled NO generation, CdS@*B. subtilis* and CdS@*E. coli* were synthesized for NO generation. Griess reagent was used to measure the NO concentration within the medium. As shown in Supplementary Fig. 7, boosted amount of NO could be produced by CdS@*B. subtilis* and CdS@*E. coli* under light irradiation. Neither CdS nor *B. subtilis* or *E. coli* alone did produce any NO under the same condition.

**Supplementary Note 3:**

**JC-1, DCFH-DA and dihydroethidium staining.** DCFH-DA staining was used to analyze the level of reactive oxygen species (ROS), which detected the brightest obvious fluorescent intensity with CCN@*E. coli* treated cells exposed under light (Supplementary Fig. 9). To investigate the PMT induced damage of mitochondria by CCN@*E. coli*, JC-1 dye was chosen as the sensor to evaluate the destruction of mitochondria. After light irradiated CCN@*E. coli* treatment, strong green fluorescence in cytoplasm and red fluorescence in cytomembrane could be observed, and this result indicated that boost amount of ROS induced. However, negligible fluorescence enhancement could be observed in other control groups. In addition, increased intracellular oxidative stress would cause irreversible mitochondria damage. Mitochondrial transmembrane potential was then analyzed by using JC-1 staining to investigate disruption and damage of mitochondria. Green fluorescence of J-monomers increased gradually, and together, red fluorescence of J-aggregates was weakened. These variations confirmed that the light irradiated CCN@*E. coli* treatment did generate NO and damage mitochondria of the 4T1 cells.

Herein, dihydroethidium, a superoxide indicator was used to measure superoxide level in 4T1 cells after treated with CCN@*E. coli*. As shown in Supplementary Fig. 9c, we found that CCN@*E. coli* + light treatment significantly enhanced the superoxide level in 4T1 cells. Whereas, neither CCN alone nor *E. coli* could induce the superoxide radical generation in 4T1 cells.

***In vitro* study of the cell apoptosis.** We evaluated the apoptosis of 4T1 cells by immunofluorescent assays and TUNEL assays (Supplementary Fig. 10). Results of immunofluorescent imaging showed that 4T1 cells had remarkably increased the expressions of Bax and active Caspase-3 after treating with CCN@*E. coli*/light. Notably, the upregulation of proliferating 4T1 cells were inhibited by CCN@*E. coli.* These results indicated that there were few apoptotic cells in control groups, and more apoptotic cells in the CCN@*E. coli*/light group. Evidenced by TUNEL staining, DNA fragmentation was observed in most cancer cells.

**Supplementary Note 4:**

**Tumor penetration of CCN@*E. coli*.** In Figure 3f and Supplementary Figure 13a, the co-localization of CCN@*E. coli* and tumor hypoxia areas was found. Besides, we also discovered that hypoxia could trigger chemotaxis of CCN@*E. coli* (Figure 3g). Then, vessels among the tumor were stained with CD31. By using immunofluorescent staining, we found that most CCN@*E. coli* accumulated outside of blood vessels (Supplementary Figure 15b), and this phenomenon suggested that *E. coli* were able to penetrate across the blood vessel wall. Even in the area that lacked of blood vessels, CCN@*E. coli* still occurred. Overall, we concluded that CCN@*E. coli* could reach to interior malignant cells which were distant from blood vessels. Considered together with its satisfactory penetrability (Supplementary Figure 15b), we believed that *E. coli* could move extravascularly through the tumor in response to hypoxia.

**Supplementary Note 5:**

**Immunological effect of PMT.** To prove this mechanism, the culture medium of PMT treated 4T1 cells was analyzed. We found that PMT treatment could trigger the secretion of HMGB1 in dying tumor cells. In this study, the HMGB1 containing medium was also co-cultured with dendritic cell (DC). After 24 h of co-incubation, the up-regulations of DC maturation markers CD80 and CD86 were observed (Fig. 5f)^2^. Thus, the purposed pathway was verified in an in vitro simulation environment. As shown in Fig 5g, after the initiation of HMGB1 mediated immune response, DC was recruited toward dying cancer cells. As illustrated in Fig. 5h, within PMT treated tumor, the ratio of mature DC-dying cancer cell distance to mature DC-living cancer cell distance was 4.5 times and 6.3 times smaller than PBS and *E. coli* group, respectively. This phenomenon indicated the initiation of anti-cancer immune responses. The Euler distance between CD8+ cytotoxicity T cells and dying cancer cells of PMT group was 2.63 times closer than the PBS group (Fig. 5i). Subsequently, as a strong evidence, the co-localization of the fluorescence from granzyme-B and active-caspase-3 was found in PMT treated tumor (Mander overlap coefficient of 0.98), which proved that some cancer cells were killed by cytotoxicity T cells, (Supplementary Fig. 21). Two days after the in vivo PMT, increased concentration of some important cytokines in serum demonstrated that PMT provoked the immune response (Fig. 5j). Overall, these results uncovered that, apart from directly generating cytotoxic NO, inducing immunogenic cell death is also an anti-cancer mechanism of PMT.

**Supplementary References**

1 Liao G. et al. Graphene oxide modified g-C_3_N_4_ hybrid with enhanced photocatalytic capability under visible light irradiation. *J. Mater. Chem.* 22, 2721-2726 (2012).

2 Vacchelli, E. et al. Chemotherapy-induced antitumor immunity requires formyl peptide receptor 1. *Science* **350**, 972-8 (2015).
