# Supplementary material for: Optically-controlled bacterial metabolite for cancer therapy
Source: Nat Commun. 2018 Apr 26;9:1680. doi: 10.1038/s41467-018-03233-9 (PMC5920064; doi:10.1038/s41467-018-03233-9)
Supplement: Supplementary file 2 — Descriptions of Additional Supplementary Files [file 41467_2018_3233_MOESM2_ESM.pdf]

Descriptions of Additional Supplementary Files

File Name: Supplementary Data 1

Description: Differential proteins in tumors after PMT treatment
